# Dispersal ability, habitat characteristics, and sea-surface circulation shape population structure of *Cingula trifasciata* (Gastropoda: Rissoidae) in the remote Azores Archipelago

**DOI:** 10.1186/s12862-021-01862-1

**Published:** 2021-06-22

**Authors:** L. Baptista, H. Meimberg, S. P. Ávila, A. M. Santos, M. Curto

**Affiliations:** 1 Institute for Integrative Nature Conservation Research, University of Natural Resources and Life Sciences (BOKU), Vienna, Austria; 2 CIBIO, Centro de Investigação em Biodiversidade e Recursos Genéticos, InBIO Laboratório Associado, Pólo dos Açores, 9501-801 Ponta Delgada, Azores, Portugal; 3 MPB-Marine Palaeontology and Biogeography Lab, Universidade Dos Açores, 9501-801 Ponta Delgada, Azores, Portugal; 4 Faculdade de Ciências da Universidade do Porto, Rua Do Campo Alegre, 1021/1055, 4169-007 Porto, Portugal; 5 Departamento de Biologia, Faculdade de Ciências e Tecnologia, Universidade Dos Açores, 9501-801 Ponta Delgada, Azores, Portugal; 6 Centro de Investigação em Biodiversidade e Recursos Genéticos, CIBIO, InBIO Laboratório Associado, Universidade do Porto, Campus de Vairão, Rua Padre Armando Quintas, no. 7, 4485-661 Vairão, Portugal; 7 MARE, Marine and Environmental Sciences Centre, Faculdade de Ciências, Universidade de Lisboa, Campo Grande, 1749-016 Lisboa, Portugal

**Keywords:** Rissoidae, Cingula trifasciata, Population structure, SSR-GBAS, Speciation

## Abstract

**Background:**

In the marine realm, dispersal ability is among the major factors shaping the distribution of species. In the Northeast Atlantic Ocean, the Azores Archipelago is home to a multitude of marine invertebrates which, despite their dispersal limitations, maintain gene flow among distant populations, with complex evolutionary and biogeographic implications. The mechanisms and factors underlying the population dynamics and genetic structure of non-planktotrophic gastropods within the Azores Archipelago and related mainland populations are still poorly understood. The rissoid *Cingula trifasciata* is herewith studied to clarify its population structure in the Northeast Atlantic Ocean and factors shaping it, with a special focus in intra-archipelagic dynamics.

**Results:**

Coupling microsatellite genotyping by amplicon sequencing (SSR-GBAS) and mitochondrial datasets, our results suggest the differentiation between insular and continental populations of *Cingula trifasciata*, supporting previously raised classification issues and detecting potential cryptic diversity. The finding of connectivity between widely separated populations was startling. In unique ways, dispersal ability, habitat type, and small-scale oceanographic currents appear to be the key drivers of *C. trifasciata*’s population structure in the remote Azores Archipelago. Dispersal as non-planktotrophic larvae is unlikely*,* but its small-size adults easily engage in rafting. Although the typical habitat of *C. trifasciata,* with low hydrodynamics, reduces the likelihood of rafting, individuals inhabiting algal mats are more prone to dispersal. Sea-surface circulation might create dispersal pathways for rafts, even between widely separated populations/islands.

**Conclusions:**

Our results show that gene flow of a marine non-planktotrophic gastropod within a remote archipelago can reveal unanticipated patterns, such that the understanding of life in such areas is far from well-understood. We expect this work to be the starting of the application of SSR-GBAS in other non-model marine invertebrates, providing insights on their population dynamics at distinct geographical scales and on hidden diversity. How transversal is the role played by the complex interaction between functional traits, ecological features, and sea-surface circulation in the population structure of marine invertebrates can be further addressed by expanding this approach to more taxa.

**Supplementary Information:**

The online version contains supplementary material available at 10.1186/s12862-021-01862-1.

## Introduction

Species’ ranges are shaped by multiple biotic and abiotic factors; however, in the marine realm, dispersal ability is one of the main factors influencing the distribution of taxa, with significant evolutionary and biogeographic implications [1, 2]. Dispersal is frequently related to the duration and behaviour of marine invertebrates’ larval stages, classified either as planktotrophic or non-planktotrophic (np), the latter comprising lecithotrophic and direct developers [3, 4]. Among the dispersal strategies reviewed by Winston [5], rafting is the most relevant mechanism followed by epibenthic, shallow-water (< 50 m depth) np-invertebrates in temperate Atlantic waters [2, 6]. Details concerning the larval development are only known for a few gastropods (e.g. [7–12]), but are unclear for most species, in particular small ones. Dispersal pathways and processes are therefore only poorly understood in many marine invertebrates. In particular, the characteristics of dispersal could play a role to explain differentiation and genetic structure in response to ecological and geographical constrains.

**Fig. 1:**
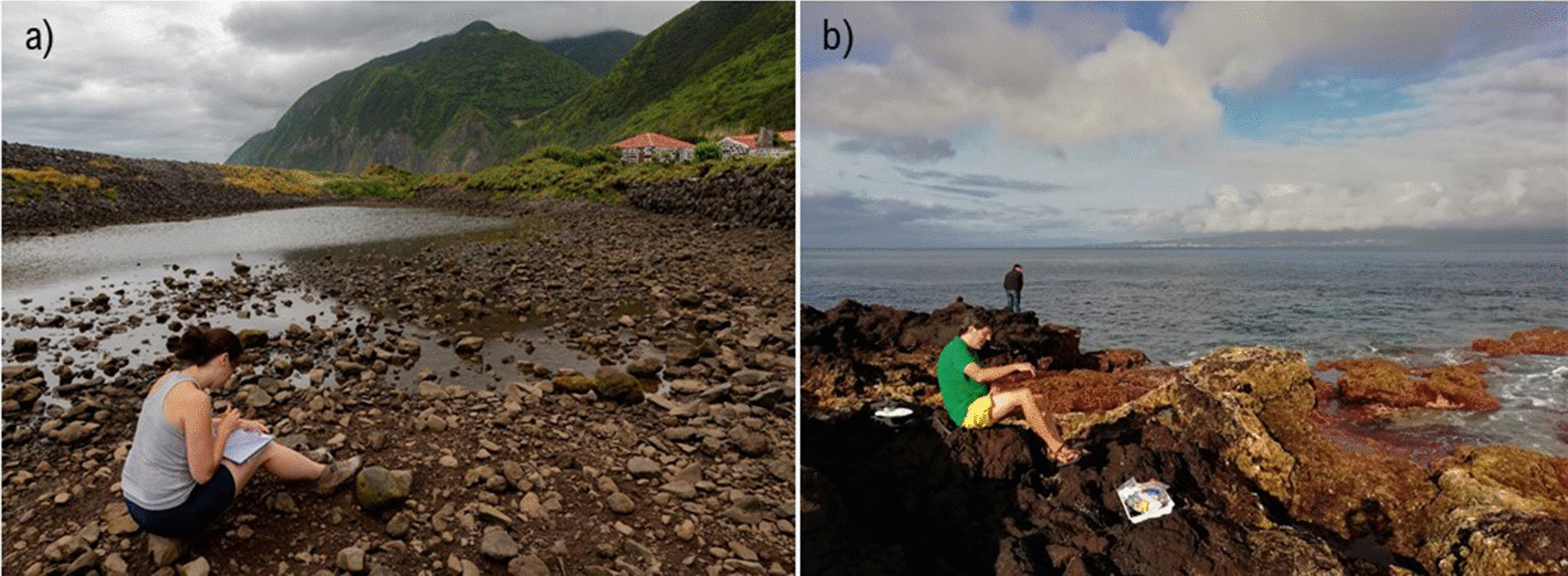
Typical intertidal habitats of *Cingula trifasciata*. **a** Enclosed gravel/boulder areas, protected from wave action; photo at Fajã da Caldeira de Santo Cristo (São Jorge Island, Azores) by Sérgio P. Ávila; **b** In the algal turf; photo at Horta (Faial Island, Azores) by Lara Baptista. Permission to publish these images was granted by the photographers and people displayed in them

Rissoidae Gray, 1847 is one of the best-known family of microgastropods. Research has been conducted over the past decades regarding distinct aspects of their biology, ecology, and evolution [10, 13–24]. The family comprises the largest number of small-sized, marine gastropod species, conspicuous around the world and with 546 species in the Atlantic Ocean and Mediterranean Sea [13, 19, 23]. Among these, *Cingula trifasciata* (J. Adams, 1800) is found throughout the Northeast Atlantic Ocean, being reported for the Azores Archipelago, Iberian Peninsula, up to the Bay of Biscay and British Isles [24–29]. This microgastropod species is commonly found at intertidal areas with low hydrodynamics and mesotidal regimes, which get exposed during low tide and submerged during high tide. A typical habitat for *C. trifasciata* is protected and enclosed gravel intertidal areas (Fig. 1a), especially beneath gravel/boulders that provides shelter and environmental stability [13, 14, 30]. The species is also present in the algal turf (Fig. 1b), which in turn provide protection to wave action, predation, and desiccation during low tide [31]. Several features of the rissoid *C. trifasciata* increase the likelihood of initiate dispersal by rafting, namely: (1) its minute size (< 5 mm); (2) its high abundance; (3) its association with intertidal habitats; and, (4) the secretion of mucus from a posterior pedal gland that allows juveniles and adult rissoids to suspend themselves from the surface film [2].

Nevertheless, *C. trifasciata* is a np-species (direct developer), laying one to four eggs in algae [13]. It is expected to be geographically restricted, as the lack of a free-swimming stage negatively affects the dispersal ability at early-life stages [6, 32, 33]. Its wide distribution across the NE Atlantic Ocean and reports on the Mediterranean Sea—Ceuta [34, 35]; Adriatic Sea [31]—challenge the expectations for a np-species [24], although examples of other np-species with wide biogeographic ranges exist (e.g. *Lasea* spp. [36], Calyptraeid gastropods [37], trochids [38]). The np nature of *C. trifasciata* suggests that long-distance dispersal strategies other than rafting are unlikely. If that is the case, surface water currents should play a major role in shaping *C. trifasciata* genetic variation. These patterns may be especially intricate in a remote volcanic oceanic island system such as the Azores Archipelago.

**Fig. 2:**
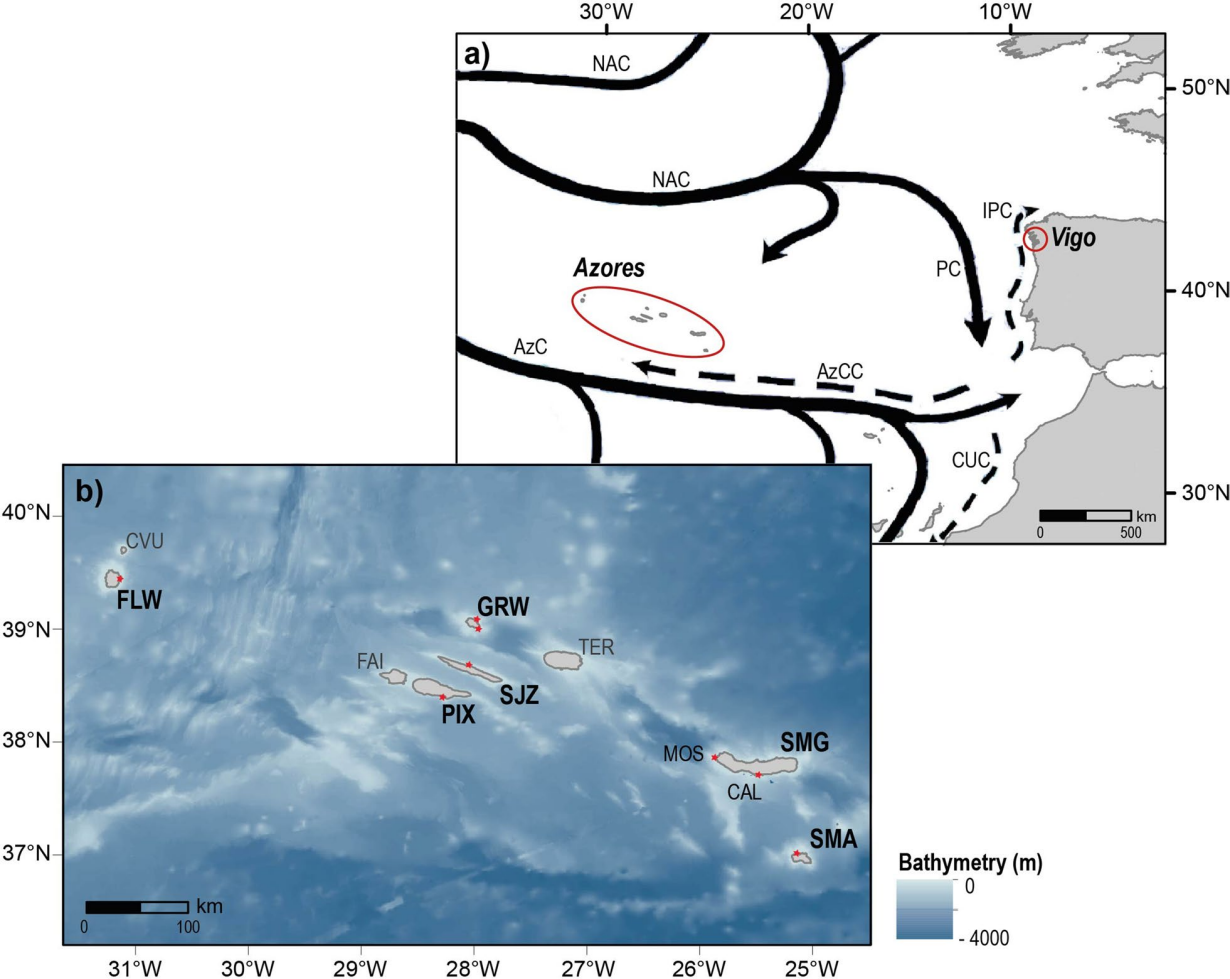
Study area in the Northeast Atlantic Ocean. **a** Geographical location of the Azores Archipelago (Portugal) and Vigo (Galicia, Spain). Major surface circulation patterns in the Northeast Atlantic Ocean are represented: AzC (Azores Current), AzCC (Azores Counter Current), CUC (Canary Upwelling Current), IPC (Iberian Poleward Current), NAC (North Atlantic Current), PC (Portugal Current). **b** Detailed geographical location of the Azorean islands. In bold are the islands sampled: SMA (Santa Maria) and SMG (São Miguel) in the eastern group; PIX (Pico), SJZ (São Jorge), and GRW (Graciosa) in the central group; FLW (Flores) in the western group. The sampling sites are identified with a red point, in São Miguel two localities were sampled: Mosteiros (MOS) and Caloura (CAL). Unsampled islands are identified: TER (Terceira), FAI (Faial), and CVU (Corvo). Coastline from the Portuguese Hydrographic Institute and bathymetry derived from GEBCO [46, 47]

The Northeast Atlantic Ocean surface circulation is characterized by a complex dynamics of the North Atlantic’s subtropical gyre (Fig. 2a). Near the Great Banks off Newfoundland, the Gulf Stream branches into the North Atlantic Current system to the north and the Azores Current south eastwards, which then flows south eastwards towards Madeira and the Gulf of Cádiz [39–41]. In this area, nine volcanic oceanic islands spread over 650 km at a west-northwest to east-southeast orientation, forming the remote Azores Archipelago, located hundreds of kilometres from other landmasses and divided in three island groups: Eastern Group (Santa Maria and São Miguel), Central Group (Terceira, Graciosa, Pico, Faial, and São Jorge), and Western Group (Flores and Corvo) (Fig. 2b). Oceanographic circulation in this region is particular: east of the islands, the Azores Current and associated front flow between 30 and 37.5°N on time scales ranging from months to decades (mean 33.9 ± 1.3°N) [42]; north of the Azores Front, and mostly at subsurface levels centred around 36°N, the Azores Counter Current circulates westward [40, 43–45].

Using molecular tools, important biogeographical questions, can be addressed, such as: (1) is there gene flow between continental and insular populations of the same species?; (2) how is the species dispersing within the archipelago, among distant populations?; (3) what are the drivers and factors influencing dispersal in the Azores? Similar questions have been posed in previous studies that intended to clarify patterns and processes affecting marine gastropods in the Azores [24, 48].

Mitochondrial markers display characteristics that make them powerful tools for the inference of molecular variability and population genetic structure, namely maternal inheritance, absence of genetic recombination, fast evolutionary rate, and increased susceptibility to the effects of genetic drift [49]. Among this type of markers, the protein-coding Cytochrome Oxidase subunit I (COI) is among the most widely used genetic marker for intraspecific analysis and population dynamics inference in animals [49, 50]. Among marine gastropods, COI provides a particularly good phylogeographic signal within the superfamily Rissooidea [16, 51, 52], to which *Cingula trifasciata* belongs. Microsatellites (SSR), due to their codominant nature, biparental inheritance, high heterozygosity, and polymorphism levels, and being multi-allelic, are more informative and powerful than other markers [53–56].

During the past decades, microsatellite sets have been developed and characterized for marine gastropods, especially for abundant, commercially interesting or threatened species (e.g. [57–64]). These sets of SSR markers appear to be skewed towards large-sized genera, such as *Nucella, Littorina*, *Buccinum*, *Haliotis* and *Concholepas*. SSR markers, sometimes coupled with other molecular markers, have mainly been used in marine gastropods for paternity studies [65–67], phylogeography and species delimitation [68, 69], population structure [70–74], as well as dispersal and connectivity associated to larval development [75–78]. Microgastropods tend to be understudied when compared to larger relatives, requiring special attention in field surveys and still associated with taxonomic classification issues [79]. This negative bias in the knowledge of microgastropods is also found in the application of molecular techniques to improve the study of population dynamics and phylogenetic questions.

In this work we investigate the processes shaping the genetic diversity and structure patterns of *C. trifasciata,* with a special focus on the influence of sea surface circulation. This was done by applying a traditional analysis of the COI molecular marker from several populations and developing a set of primers for microsatellite markers for this non-model species, using the SSR-GBAS approach, a method that relies on amplicon sequencing with second generation sequencing techniques to determine genotypes at microsatellite containing loci. By doing so, we aim to increase the current knowledge of the widespread rissoid *C. trifasciata*, as well as to clarify the intraspecific genetic structure and population dynamics of this species in the NE Atlantic Ocean, with an emphasis on its behaviour in the remote Azorean islands. With this study, we intend to understand the role of habitat and small-scale surface currents among islands in shaping the genetic structure in a remote archipelago.

## Results

### Sequence data and genetic analyses

The COI dataset comprised a total of 75 sequences, with seven to 14 individuals per population. A total of 44 haplotypes were distinguished in the 658 bp alignment, none shared between populations from the Azores and Vigo. Among Azorean haplotypes, the estimates of evolutionary divergence ranged from 0.2 to 2.6% (see Additional file 1: Table S2). Unexpected high levels of divergence (~ 2%) within the populations at Graciosa and Santa Maria are detected, but a manual check of the chromatograms ensured that the reported variation is real and not a consequence of misreads. Differences between the two populations at Graciosa Island seem to be negligible, thus being considered a single population for interpretation purposes. The lowest inter-locality differentiation levels are found in the comparisons of haplotypes from the central group, namely São Jorge, Pico, Graciosa islands, and some haplotypes from the western locality Mosteiros in São Miguel island. Haplotypes from Vigo show low within population divergence (average 0.23%), but considerably high differentiation in relation divergence to the Azorean haplotypes, ranging from 3.6 to 5%.

**Fig. 3:**
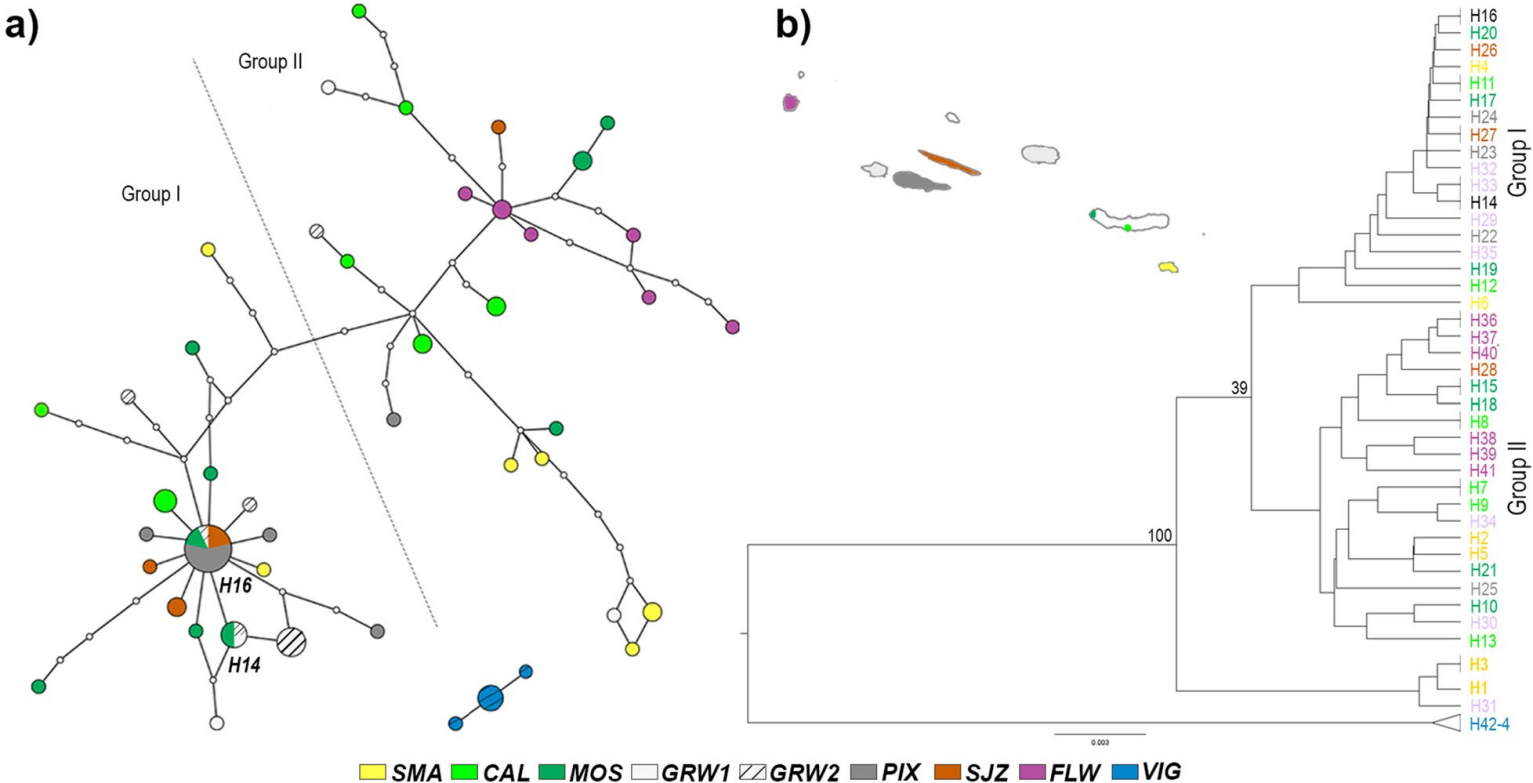
Distribution of mitochondrial haplotypes of *Cingula trifasciata*, based on 75 COI sequences/44 haplotypes. **a** Haplotypic network of nine colour-coded populations, at 95% parsimony connection limit. Size of the circles proportional to the frequency of each haplotype; small uncoloured circles represent non-observed haplotypes; each line connecting haplotypes represents a single mutational change. Network obtained with TCS v1.21 [81] and tcsBU [82]; **b** UPGMA tree [83], as implemented in Geneious 8.1.9 [84] and considering 1000 replicates for the bootstrap inference. Haplotypes coloured according to the population in which they are detected, black indicates haplotypes present in more than one population. Geographical representation of Azorean islands/localities colour-coded; distances among island groups not to scale

**Fig. 4:**
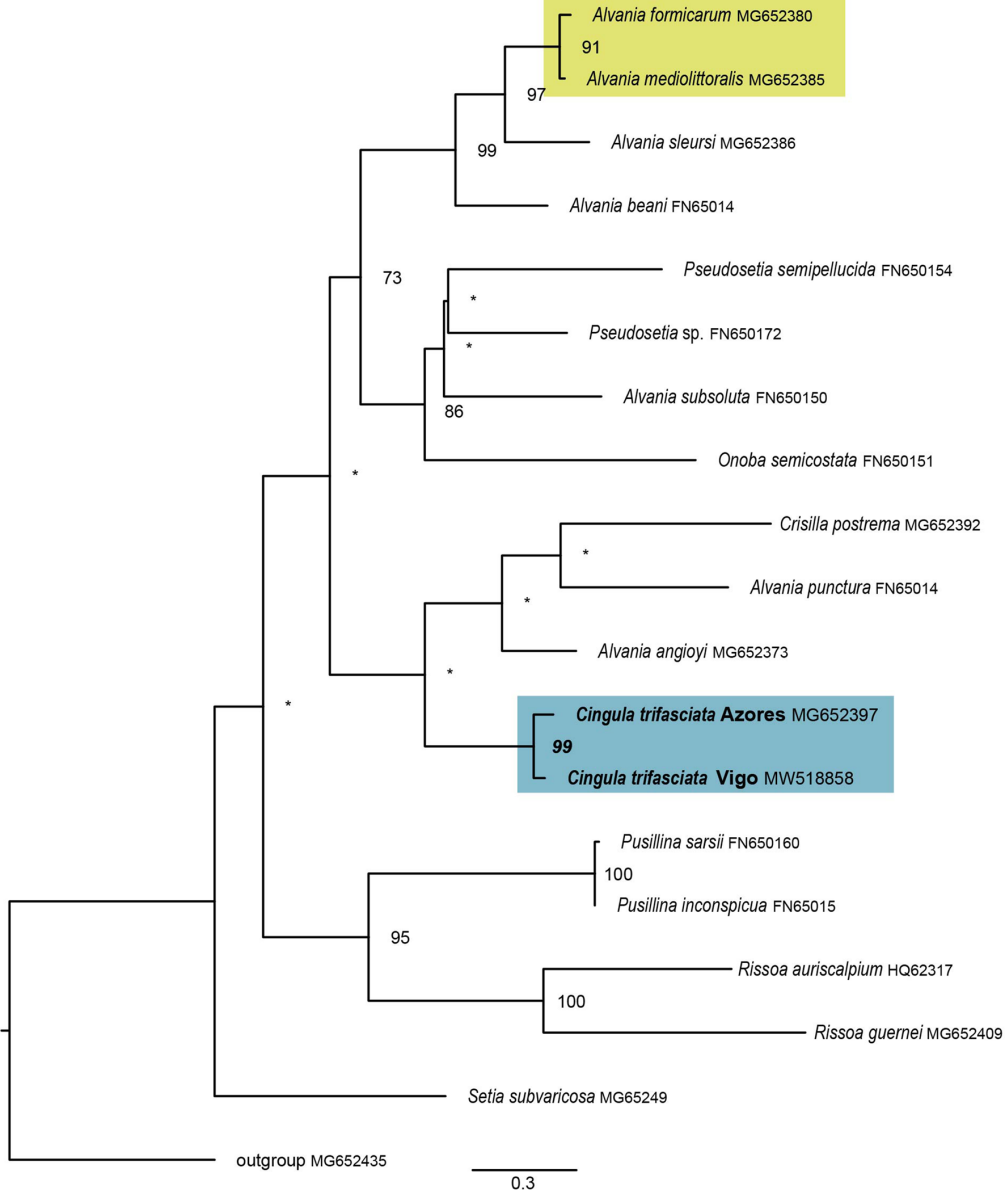
Maximum Likelihood phylogenetic tree of 17 species of the family Rissoidae. Inferred with RAxML v8.2.7 [85] under the GTR model of substitutions. Highlighted in blue are *Cingula trifasciata* from the Azores and Vigo; in yellow, the recognized species *Alvania formicarum* and *A. mediolittoralis*. Asterisks (*) indicate support values below 70%. Accession numbers provided in the tree

The distribution of mitochondrial haplotypes (Fig. 3) reflects the estimates of evolutionary divergence, with congruent relationships inferred by the TCS network and UPGMA tree. Only two haplotypes are shared by two or more populations (H14 among Mosteiros (São Miguel Island) and both populations of Graciosa Island; H16 among Mosteiros, Graciosa, Pico, and São Jorge), the remaining exclusive to the population in which they are found. This level of genetic differentiation and uniqueness of the mitochondrial set is observed even within the same island, as Mosteiros and Caloura (São Miguel Island) share no haplotypes, although the first shares haplotypes with more distant central islands. The closely related haplotypes from Pico, São Jorge, and Graciosa form one major star-shaped group in the TCS network (Fig. 3a) that also includes one haplotype exclusive to Santa Maria Island (H4), another exclusive to Caloura (H11) and several from Mosteiros. The remaining haplotypes form a disperse second group, connected by several inferred mutation steps, which comprises haplotypes from the eastern (Santa Maria and most Caloura and Mosteiros’ haplotypes from São Miguel) and western (Flores) group of Azorean islands. Haplotypes 25 (Pico) and 28 (São Jorge) were found in this second group of the network, while haplotypes from Graciosa, Mosteiros and Caloura can be found in both groups. Haplotypes from Vigo (H42-44) form an isolated cluster, not connected to the Azorean haplotypes. The reconstruction of a Rissoidae phylogenetic tree (Fig. 4) allowed to clarify the divergence levels within the family and to ascertain the position of *C. trifasciata* from the Azores and Vigo. The separation of the samples from the mainland and the islands is well-supported (99%) in the phylogeny. A BLASTN search [80] of VIG sequences revealed 100% cover and 95–96% identity with several *C. trifasciata* haplotypes from the Azores, but 99–100% identity with a shorter Galician sequence deposited at the database (KU695304; 55% query cover).

Pairwise F-statistics between populations were estimated for the COI dataset (cf. Additional file 1: Table S3 for details). Comparatively high and significant levels of differentiation, over 0.8, were inferred between Vigo and Azorean populations. Among Graciosa, Pico, and São Jorge Islands, the F_ST_ levels were low and non-significant, whereas Mosteiros (São Miguel Island, eastern group) shows only significant differences with Graciosa and Pico from the central group. The analysis revealed significant and high F_ST_ values in the pairwise comparisons with Flores Island. In comparison with the remaining estimates, the pairwise comparisons involving Flores, Santa Maria, and Caloura retrieved higher F_ST_ levels, suggesting higher isolation of these populations.

### SSR marker discovery

For the SSR marker discovery, the MiSeq runs for the two samples produced 766,960 and 1,382,293 paired reads, respectively for PSM4350 and FSC5131. A total of 445,578 and 248,419 reads, which passed the quality control and merging steps were screened for SSR motifs. Of these, respectively, 450 and 8,673 reads containing SSR motifs complied with the criteria defined for the SSR search pipeline. These comprised 203 di-, 123 tri-, 107 tetra-, and 17 pentanucleotide repeats for PSM4350; and 2168 di-, 1477 tri-, 1303 tetra-, and 254 pentanucleotide repeats for FSC5131. A total of 42 primer pairs for *C. trifasciata* were designed only from sequences containing penta- and tetranucleotide repeats. Eleven out of the 42 designed primers failed to amplify in the single PCR test phase. The remaining primers were included in 4 multiplex primer mixes and are provided in Additional file 1: Table S1.

### SSR data analysis and SSR-GBAS genotyping

For the SSR-GBAS genotyping, a total of 2,543,202 paired reads were produced, which were reduced to 1,008,047 after quality control, merging and primer demultiplex steps. Raw reads can be accessed through the BioProject PRJNA702169. The number of reads per marker ranged from 1,794 to 121,460, with CT42_TAAA being the one with highest number of sequences. Regarding the number of sequences per sample, it varied between 38 (PS4380) and 56,441 (CAL5184). After filtering the matrix and exclude the samples with more than 60% missing data across all markers, the dataset was reduced to seven individuals from Santa Maria, nine from Caloura and two from Mosteiros (São Miguel), six from São Jorge, 11 from Pico, five from Graciosa, one from Flores, and three from Vigo (Galicia, Spain). As only one sample was successful in SSR-GBAS genotyping, the population from Flores island was excluded from further analyses. Although they had been successfully amplified in the single PCR test, the following markers were excluded due to failure in the multiplex step or Illumina MiSeq, resulting in data unfit for further analysis or missing data: CT6_TTGT and CT17_TTTG were excluded due to non-specific amplification; CT1_TGTT, CT3_GAATA, and CT10_GTTT were excluded for yielding over 60% missing data. Thus, a total of 26 markers and 43 samples were suitable for population genetic analyses of *C. trifasciata*.

### Population structure analyses

#### Genetic diversity measures

In the WAI (Whole Amplicon Information) dataset, the markers analysed had between 6 and 41 alleles (Additional file 1: Fig. S1a), which can be found in GenBank Database [86] under the accession numbers MW623085-363. The polymorphic information content (PIC) per locus ranged between 0.43 and 0.954, with only 4 out of 26 markers displaying PIC values below 0.50. Although monomorphic markers for the complete WAI dataset were not detected, some of the markers were monomorphic in some populations (Table 1): CT23_TTTA in Caloura; CT24_CAAA in Graciosa; CT31_AATA in Graciosa; CT38_TCAT in Caloura; plus eight monomorphic markers in Vigo population (data not shown). Not accounting monomorphic markers across the studied populations, the complete WAI dataset showed *Ho* varying from 0.235 to 0.921 and *He* from 0.448 to 0.956. Within each of the five populations in study, with five or more individuals (Additional file 1: Fig. S1b), the number of alleles (Na) ranged from 3.346 (São Jorge) to 4.538 (Santa Maria) all with a frequency over 5%, except in Pico. The number of effective alleles (Ne) was lower to Na in all populations, ranging from 2.357 (Pico) to 3.317 (Santa Maria). *Ho* and *He* followed the same trend across all populations when analysing the dataset per population, with *Ho* levels varying between 0.394 (São Jorge) and 0.620 (Santa Maria), and *He* between 0.498 (São Jorge) and 0.623 (Santa Maria). Private alleles were detected in all populations.

**Table 1:** Estimates of Hardy–Weinberg Equilibrium tests, frequency of null alleles and Wright’s Fixation index

	Santa Maria			Caloura			Graciosa				Pico		São Jorge		
	HWE Test	f(Null)	F_IS_	HWE Test	f(Null)	F_IS_	HWE Test	f(Null)	F_IS_	HWE Test	f(Null)	F_IS_	HWE Test	f(Null)	F_IS_
CT11_GTTT	0.296	0.189	0.378	0.284	0.001	0.357	0.164	0.000	0.565	0.218	0.119	0.371	0.828	0.001	0.077
CT13_GTTG	0.787	0.000	0.091	0.967	0.000	− 0.185	0.821	0.001	− 0.304	0.567	0.001	0.263	0.446	0.000	− 0.212
CT14_CAGA	0.784	0.000	0.103	0.223	0.001	− 0.091	0.577	0.072	0.000	0.562	0.000	0.083	0.008**	0.420	1.000
CT15_TTTG	0.208	0.157	0.138	0.120	0.042	0.385	0.256	0.153	0.333	0.740	0.000	− 0.100	0.804	0.001	− 0.111
CT16_TTTG	0.904	0.000	− 0.321	0.987	0.000	− 0.091	0.804	0.000	− 0.111	0.218	0.001	0.371	0.341	0.001	0.429
CT18_AAAG	0.659	0.000	− 0.167	0.093	0.066	0.032	0.958	0.000	− 0.176	0.987	0.000	− 0.183	0.007**	0.251	0.429
CT19_GACA	0.907	0.000	− 0.200	0.388	0.081	0.091	0.704	0.000	− 0.250	0.113	0.063	0.173	0.824	0.001	− 0.091
CT21_CAAA	0.594	0.000	− 0.014	0.143	0.153	0.123	0.475	0.000	0.143	0.725	0.000	− 0.111	0.775	0.000	− 0.143
CT22_ACAG	0.435	0.000	0.082	0.777	0.000	− 0.400	0.353	0.001	0.040	0.786	0.000	− 0.158	0.775	0.000	− 0.143
CT23_TTTA	0.682	0.001	− 0.371	*mono*	0.001	−	0.351	0.153	0.467	0.01**	0.164	0.807	0.034*	0.082	0.429
CT24_CAAA	0.540	0.000	− 0.373	0.480	0.001	− 0.011	*mono*	0.001	–	0.763	0.030	0.091	0.025*	0.001	1.000
CT26_TGTT	0.270	0.138	0.417	0.326	0.053	0.024	0.120	0.269	0.167	0.724	0.001	− 0.077	0.931	0.000	− 0.231
CT28_CTGT	0.995	0.000	− 0.296	0.524	0.000	− 0.043	0.704	0.000	− 0.250	0.791	0.000	− 0.245	0.244	0.000	0.143
CT29_AAAT	0.166	0.000	0.111	0.666	0.000	0.217	0.062	0.290	1.000	0.958	0.000	− 0.132	0.125	0.269	0.412
CT30_TGTC	0.138	0.000	0.125	0.207	0.197	0.652	0.393	0.000	0.000	1.000	0.000	− 0.200	0.132	0.001	0.231
CT31_AATA	0.270	0.001	0.417	0.850	0.001	− 0.067	*mono*	0.001		0.774	0.001	− 0.266	0.014*	0.251	1.000
CT32_TTTG	0.188	0.000	− 0.463	0.470	0.000	0.000	0.764	0.000	− 0.250	0.541	0.000	− 0.176	0.666	0.000	0.217
CT33_CATT	0.119	0.001	0.054	0.051	0.001	0.576	0.861	0.000	− 0.333	0.002**	0.307	1.000	0.112	0.001	0.333
CT34_ACCA	0.112	0.290	1.000	0.692	0.000	0.143	0.079	0.001	0.032	0.461	0.000	0.067	0.414	0.001	− 0.333
CT35_TTTG	0.511	0.000	− 0.089	0.166	0.047	0.265	0.152	0.223	0.500	0.650	0.000	0.210	0.999	0.000	− 0.263
CT37_AAAT	0.126	0.042	0.408	0.865	0.000	− 0.220	0.958	0.000	− 0.176	0.376	0.089	0.267	0.025*	0.001	1.000
CT38_TCAT	0.814	0.000	− 0.120	*mono*	0.001	–	0.847	0.000	0.063	0.001**	0.000	0.057	0.704	0.000	− 0.250
CT39_CATT	0.451	0.143	− 0.250	0.752	0.001	− 0.167	0.891	0.001	− 0.471	0.054	0.000	− 0.618	0.216	0.251	− 0.111
CT40_ATCC	0.272	0.000	− 0.235	0.549	0.000	− 0.200	0.135	0.000	− 0.212	0.131	0.119	0.436	0.062	0.082	0.538
CT42_TAAA	0.471	0.000	− 0.273	0.804	0.000	− 0.159	0.172	0.000	− 0.724	0.461	0.000	− 0.222	0.824	0.001	− 0.091
CT5_TGAA	0.637	0.000	− 0.333	0.046*	0.333	1.000	0.963	0.000	− 0.333	0.850	0.000	− 0.067	0.572	0.001	− 0.600

Analysis per marker and population with at least five individuals. Significant (* p < 0.05, ** p < 0.01) deviations to HWE highlighted in bold. Monomorphic loci (*mono*) and loci which completely failed genotyping (*all null*) also depicted

Tests for Hardy–Weinberg Equilibrium (HWE; Table 1) per markers and population revealed deviations at one locus in Caloura (CT5_TGAA) and three loci in Pico (CT23_TTTA, CT33_CATT, CT38_CATT). The potential phenomena causing it were further evaluated with estimates of null alleles’ frequency and Wright’s Fixation index (F_IS_). Errors in the assignment of genotypes causing deviations to HWE were discarded after checking the markers plots generated after the Allele Length calling script. The deviations were either caused by the dominance of null alleles (CT5_TGAA in Caloura) inferred from FreeNA and checked in the original matrix, excess of homozygotes indicated by F_IS_ (CT33_CATT in Pico), or both (CT23_TTTA in Pico). A total of six out of 26 markers deviated from HWE in the population from São Jorge (CT14_CAGA, CT18_AAAG, CT23_TTTA, CT24_CAAA, CT31_AATA, CT37_AAAT) due to excess of homozygotes suggested by the F_IS_ levels, which led to higher frequencies of null alleles despite the absence of real null alleles. In Vigo population (data not shown), three loci failed to amplify (CT23_TTTA, CT30_TGTC, and CT5_TGAA) resulting in null alleles across all the samples of this population; the negative values of F_IS_ in the several loci suggest an excess of heterozygotes in this population.

### Genetic and spatial structure analyses

**Fig. 5:**
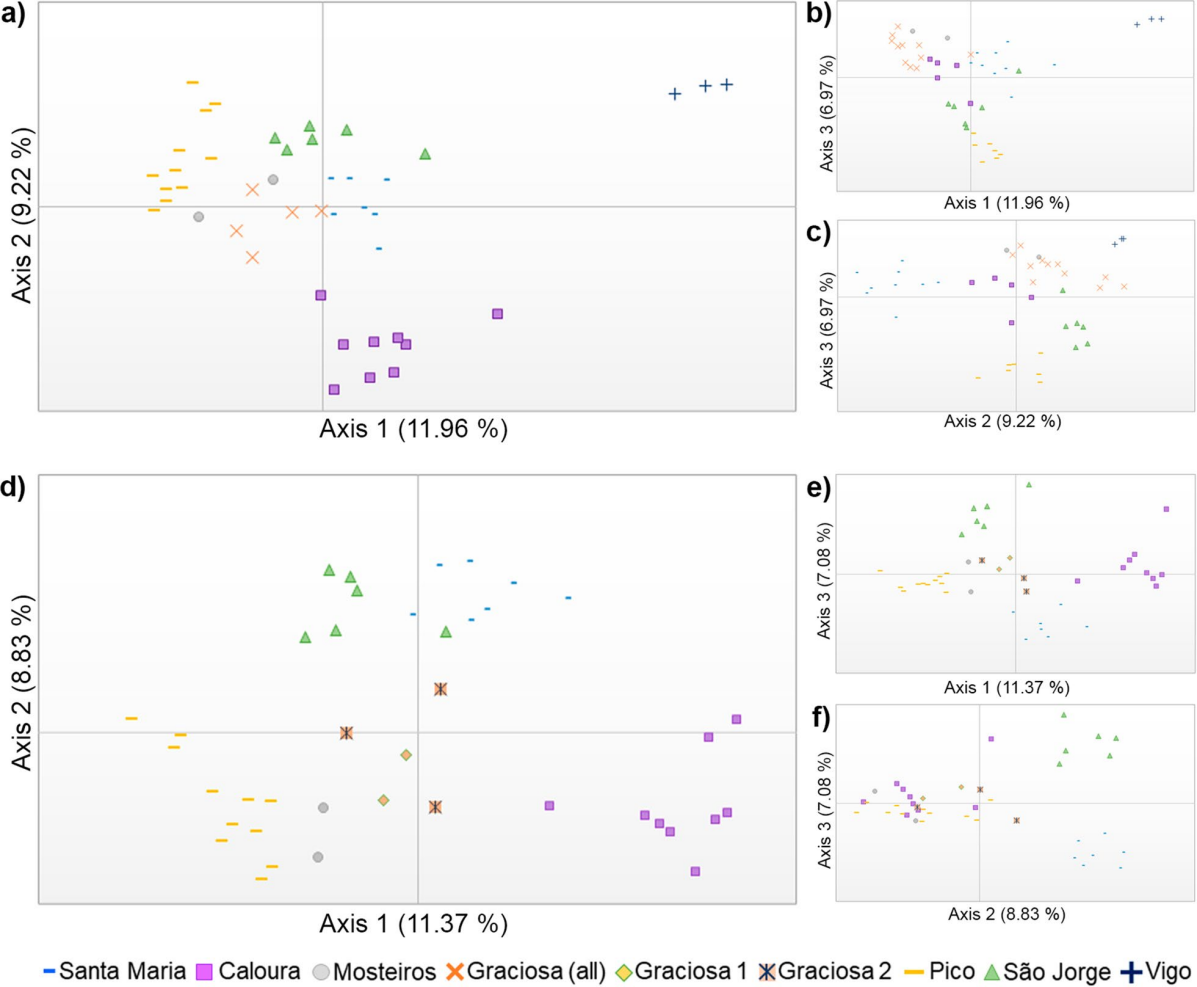
Principal coordinates analysis from complete WAI dataset of *Cingula trifasciata* (**a**–**c**) and Azorean localities (**d**–**f**). **a** Variance explained by axis 1 (11.96%) and axis 2 (9.22%); **b** Variance explained by axis 1 (11.96%) and axis 3 (6.97%); **c** Variance explained by axis 2 (9.22%) and axis 3 (6.97%); **d** Variance explained by axis 1 (11.37%) and axis 2 (8.83%) for the Azorean localities; **e** Variance explained by axis 1 (11.37%) and axis 3 (7.08%), for the Azorean localities. **f** Variance explained by axis 2 (9.22%) and axis 3 (6.97%), excluding Vigo population. PCoAs conducted as implemented in GenAlEx v6.5 [87, 88]; populations in study are coded by colour and icon shape

The PCoA analysis of the complete WAI dataset (Fig. 5a–c) revealed a clear distinction of populations from the Azores Archipelago (Santa Maria, Caloura, Mosteiros, Graciosa, Pico, São Jorge) and the one from Vigo (Galicia, Spain). The repetition of the PCoA without Vigo population allowed to clarify the similarities among Azorean populations (Fig. 5d–f), each forming smaller, distinct subclusters. In both dimensions, Mosteiros (São Miguel) is positioned between the populations from Graciosa or Pico, whereas Pico and Caloura show considerable dispersion along all the axis. Except for Mosteiros’ population, the eastern populations seem to be more divergent when compared to the remaining.

**Fig. 6:**
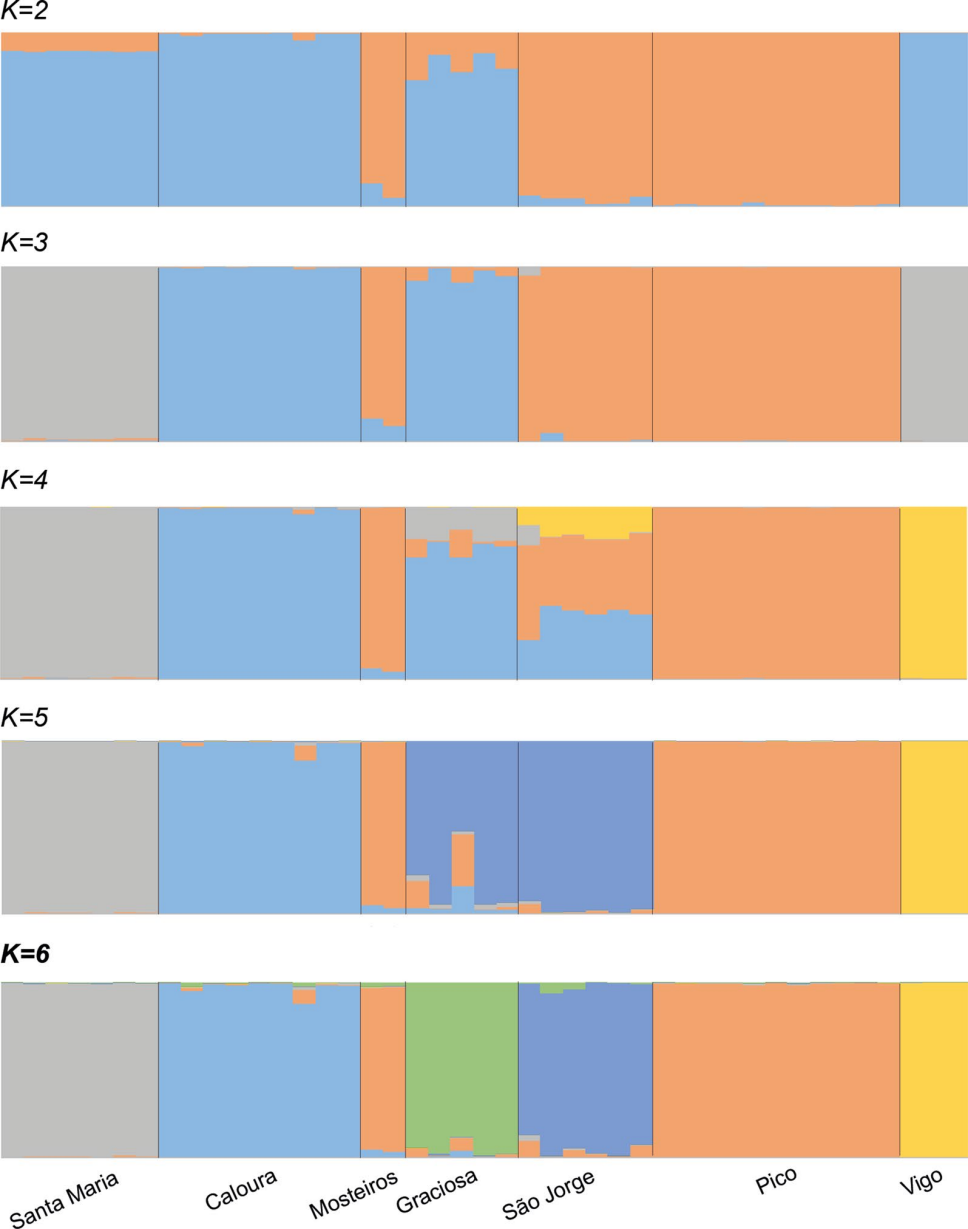
Genetic structure analysis of the complete WAI dataset of *Cingula trifasciata*. Inferred with STRUCTURE v2.3.4 [89, 90] and reporting the results from K = 2 up to the optimal K = 6

The STRUCTURE analysis was congruent with the PCoA results, with K-value of 6 estimated as optimal (Fig. 6), separating each population individually except for Pico and Mosteiros, which cluster together. At this K-value, Pico, Santa Maria, and Vigo populations each form a well-defined cluster, agreeing with the pattern retrieved from the PCoA. We have also considered lower K-values to evaluate potential correspondence between the hierarchy cluster and geographical distribution of the populations studied (Fig. 6). At K = 2, Vigo shares similar patterns of variation with the Azorean populations of Caloura and Santa Maria, whereas the second cluster is dominated by Pico and São Jorge individuals. The remaining populations, located in between these groups, show a considerable degree of admixture. At K = 3, Santa Maria and Vigo are assigned to one new cluster, while the other two clusters show evidences of admixture in some populations. Vigo forms a new cluster when K = 4 and, finally, at K = 5 it is assigned to an isolated cluster with no signal in other populations. From K = 3 to K = 5, the pattern roughly corresponds to a gradual transition of clusters from West to East. The assignment of Graciosa to a new cluster only when K = 6 falls out of this pattern, as does the unexpected placement of Mosteiros samples in the cluster together with Pico. For K-values over 7 (cf. Additional file 1: Fig. S2), cluster assignment becomes increasingly unclear for Azorean populations, and no further population sub-structure is inferred; Vigo population remains isolated.

The AMOVA analysis (Table 2), performed among regions (Azores and Vigo) and populations, revealed that 25% of the variation in the complete WAI dataset is found among regions, whereas the differentiation among populations accounts for 13% of the overall diversity. Regarding the F-statistics, a significant (p < 0.01) F_ST_ value of 0.385 was estimated for the overall WAI dataset, whereas the pairwise F_ST_ estimates for populations are detailed in Additional file 1: Table S4 (cf. Figure 8 for a summary). As expected, the highest differentiation is detected between Vigo and all the Azorean populations, with F_ST_ values over 35% in all pairwise comparisons. The lowest F_ST_ values are observed in Mosteiros vs. Pico (0.110), Mosteiros vs. Graciosa (0.119), and Graciosa vs. Pico (0.122), in congruence with the previous results of PCoA and STRUCTURE, suggesting gene flow between these populations.

**Table 2:** Hierarchical analysis of Molecular Variance among *Cingula trifasciata*

Source	*df*	*SS*	*MS*	*Est. Var*	*%*
Among regions	1	58.016	58.016	3.269	**25**
Among populations	5	160.353	32.071	1.714	**13**
Among individuals	36	362.468	10.069	2.104	16
Within individuals	43	252.000	5.860	5.860	45
Total	85	832.837		12.947	100

Analysis performed among populations and among geographical regions (Azores, Portugal vs. Galicia, Spain), with 999 permutations

## Discussion

With this work we address biogeography and the processes shaping the genetic structure and evolutionary dynamics of *Cingula trifasciata* in the NE Atlantic Ocean, with a special focus in the Azores Archipelago. The results indicate a divergence between insular and continental populations of this microgastropod and the existence of genetic structure within the Azores Archipelago on a small spatial scale. This indicates dispersal barriers for the non-planktotrophic species that could form the starting point of population differentiation. To our knowledge, this is the first time that the SSR-GBAS protocol generates a numerous set of de novo SSR primers for a marine microgastropod. Comparing to previous studies in other marine invertebrate taxa, a higher number of loci is herewith amplified contributing to an informative dataset even with a low number of individuals. Thus, this methodology has the potential to be applied in similar study systems, increasing the number of markers successfully developed for marine taxa. These topics will be further discussed in the next sections.

### A de novo SSR primer dataset for *Cingula trifasciata*

In this study, a total of 26 SSR loci of *C. trifasciata* were developed and characterized and genotyped using sequence information of Illumina MiSeq according to the SSR-GBAS methodology described earlier [91–93]. Second-generation sequencing technologies revolutionized the discovery and genotyping of microsatellites, solving technical drawbacks posed by the traditional genotyping techniques [56, 92, 94–97]. The application of next-generation sequencing (NGS) to non-model species, to which a reference genome is usually not available, is attained by downsizing approaches for the analysis of a small subset of loci, ultimately contributing to the development of genotyping by sequencing [56, 97]. The approach is frequently used in population genetics studies, detecting more microsatellite markers per sample and unique alleles in a fast and cost-effective way [56, 91, 92, 96–98]. Due to its characteristics, amplicon sequencing increases the statistical power even with a low number of sequenced markers and the resolution of detailed population genetic structure, suitable even in large-scale de novo population genetic studies in non-model species [56, 94, 97]. Moreover, this approach allows to obtain the complete sequence of the loci analysed, including the flanking region, the repetition motif, and potential SNPs (Single Nucleotide Polymorphism), overcoming homoplasy typical of SSR studies [92, 96, 98]. The recent SSR-GBAS (Simple Sequencing Repeats—Genotyping by Amplicon Sequencing) workflow calls alleles based on the whole amplicon information (WAI), including allele length (AL) and SNPs. It results in a higher number of alleles recovered, marker variability, information content, and lower influence of homoplasy, which together provide a better resolution of the SSR dataset. A script for analysis of data was provided by de Barba et al. [96] and another later described by Curto et al. [92]. Relying on approaches suggested by Illumina, the SSR-GBAS methodology follows a cost-effective optimization of a high level of multiplexing, up to 10 markers. The results are easily reproduced and analysed resorting to bioinformatic tools, allowing the automation of allele calling and reduction of traditional SSR artefacts. The SSR-GBAS method is useful for the study of small-scale systems, non-model organisms, and specific research questions, yielding potential to become an important tool to be incorporated in long-term screening projects and meta-analysis, along with data from other sources/teams, comparable to phylogenetic data collections. The SSR-GBAS methodology has been described in detail by [91–93] and summarized herewith.

In congruence with the previous studies using SSR-GBAS, a set of highly informative markers was obtained for the non-model, marine microgastropod, with moderate to high PIC values according to the classification scheme [99]. Only loci with penta- and tetranucleotide repeats were included to reduce the complexity of PCR and artefacts that interfere with the call of alleles [96]. Following this approach, allele call could be done unambiguously with no need to include manual edits after visual control of genotypes [92]. Dinucleotides more frequently produce stutter bands that make allele determination difficult and, in the case of SSR-GBAS, additionally result in a higher error rate in the determination of single nucleotide polymorphisms, when alleles that differ by one repeat unit contain a SNP [92, 100, 101]. The additive signal of allele and stutter influences the base frequency at the SNP position. In our case, the penta- and tetranucleotide repeat loci comprised a high amount of information so the inclusion of the shorter repeat unit motifs was not necessary.

In addition to being highly informative, this newly generated set of SSR markers retrieved overall diversity parameters consistent with values reported for other marine gastropods [64, 76, 77]. Deviations from HWE in some populations – Caloura, Pico, São Jorge, and Vigo –, are frequently reported in marine invertebrates [70, 72, 73, 102]. Reproductive patterns have been suggested to contribute to the excess of homozygotes in marine invertebrates [73, 103]. *C. trifasciata* possesses np-larvae with low dispersal ability, which contributes in one hand to the high genetic structure detected but also to homozygote excess in some populations. Nevertheless, this should not affect the biogeographical interpretations regarding the Azorean populations, as their prevalence in other gastropod species has been shown to not influence the population structure inferred (e.g. [68, 73]). High variability in the flanking regions might contribute to the inference of null alleles in the Vigo population [104]. Our results suggest that Azorean and Vigo populations constitute distinct evolutionary lineages, thus the specificity of the SSR primers, designed from Azorean samples, might be lower for individuals from Vigo. Therefore, the application of this set of SSR primer in Vigo’s individuals is comparable to a situation of cross-species amplification, with the potential to negatively affect the success of the protocol in the related species [92, 105].

Although laboratory and analytical methodologies have developed fast in the past decades, molecular markers for molluscs are scarce and mostly based on traditional methods, as microsatellites and allozymes [73, 76, 106], also as a consequence of the problems arising during DNA extraction of molluscan tissues [107]. The combination of markers with different information content has been shown to improve the sensitivity of population genetic inferences [108]. However, the few datasets so far available do not truly represent the genetic and geographic differentiation in marine gastropods, and the lack of widely used molecular markers hampers comparative analyses of markers’ suitability in a study system [109]. The development of de novo molecular markers, ideally transversal to related taxa, is urgently needed to increase our understanding on connectivity in the marine realm and evolution of invertebrates with different ecological and biological characteristics. SSR-GBAS methodology can be an important tool to achieve these goals, being easy to implement and fast to develop, as shown here with *C. trifasciata* [91, 92]. Datasets generated with this methodology, comprising variability from the repetition motif and SNP in the flanking region, are informative in distinguishing evolutionary entities. In understudied groups, as marine invertebrates, this type of markers might even be useful to uncover cryptic patterns of diversity, as detected in this work and further discussed in the next section.

### Differentiation between insular and continental populations

The analysis of the mitochondrial marker COI of *Cingula trifasciata* revealed differentiation between samples from the Azores Archipelago and Vigo (Galicia, Spain). A complete separation of the Azorean and Vigo networks at 95% connection limit (Fig. 3) has been previously pointed as indicative of deep differences, even considered by some as different species [110]. Divergence levels between haplotypes from the Azores and Vigo ranged from 3.6 to 5% (cf. Additional file 1: Table S2) and this distinction becomes evident at a phylogenetic tree (Fig. 4). Within the superfamily Rissooidea, to which *C. trifasciata* belongs, the COI marker is an adequate diagnostic tool for species status by its strong and reliable phylogeographic signal [52]. We have further assessed evolutionary divergence levels of COI within the family Rissoidae, ranging from 2.9 to 19.9% within the several genera analysed (Additional file 1: Table S5). The divergence between *C. trifasciata* Azores and Vigo falls within this interval, surprisingly high for conspecific individuals. A similar level of differentiation (2.9%) occurs between *Alvania mediolittoralis* (Gofas 1989) and *A. formicarum* (Gofas 1989), two recognized species occurring in the Azores Archipelago with distinctive morphological characters. The inclusion of these *Alvania* species in a calibrated molecular phylogeny of Rissoidae has shown them to have diverged quite recently, at about 0.36 million years (Ma), within a range uncertainty of 0.12–0.61 Ma [24], explaining the relatively low divergence levels. Following this rationale, we propose that a divergence event is currently acting on *Cingula trifasciata,* causing a split between the evolutionary lineages from the Azores and Vigo. The differentiation between insular and mainland (Vigo) populations is supported by the COI and WAI datasets, and evident both in clustering analyses (Figs. 5, 6). In the STRUCTURE analysis, samples from Vigo cluster together with no signs of admixture even when K = 2, and at K = 4 it forms an individual cluster, not admixed in other populations. The maintenance of this pattern and absence of further sub-structuring for K > 6 supports the uniqueness of Vigo’s samples relative to Azorean populations.

Although true allopatric isolation in the marine realm is difficult to accomplish, the high distances across deep-water and unsuitable habitats between locations could hamper dispersal and reduce gene flow between populations though to have similar ecological requirements and means of dispersal [2, 19, 111]. Lower dispersal leads to the accumulation of genetic variants exclusive to each population and perhaps partial isolation of the insular populations in relation to the mainland [111], here reflected in the genetic differences in the COI and SSR datasets. This scenario of insular isolation is not exclusive to *C. trifasciata*. Differentiation of Azorean populations, often together with high intra-archipelagic diversity levels, have been described for other marine organisms as the gastropod genus *Patella* [112] and several fishes [113–118], but also for terrestrial organisms (e.g. *Nyctalus azoreum* [119, 120]*;* starlings [121]).

**Fig. 7:**
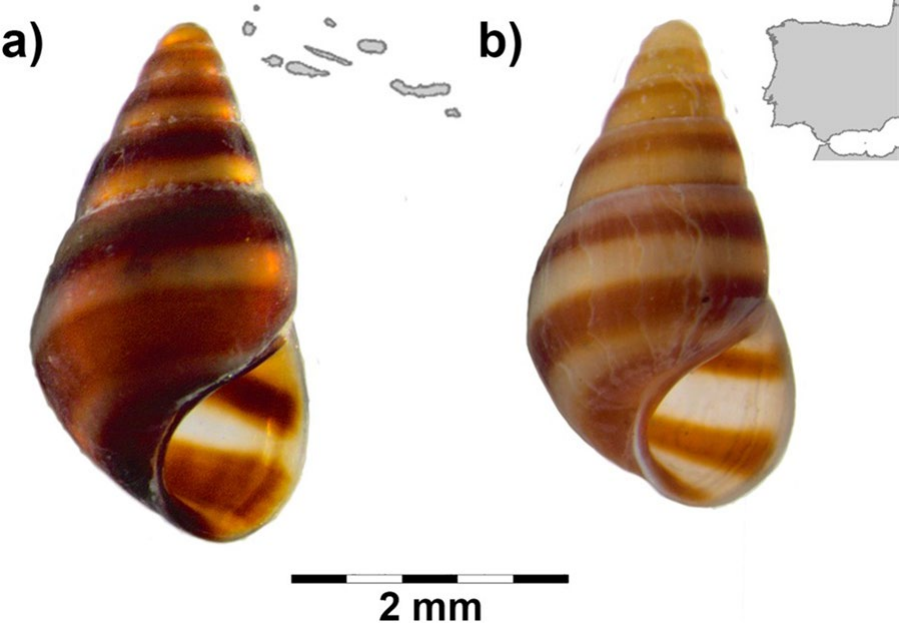
*Cingula trifasciata* specimens from insular and mainland populations. **a** Cerco da Caloura, São Miguel Island, Azores (PT-CAL3; DBUA 1474) and **b** Vigo, Galicia, Spain (ES-VIG4400)

Morphological differences between Azorean and Iberian individuals of *C. trifasciata* have previously been reported [14], in easily observable characters (e.g. broader brown bands on the whitish shell; cf. Figure 7) and in detailed observations of the tentacles and snout. Doubts regarding the assignment of Azorean *C. trifasciata* to the European taxon raised two decades ago are now supported by molecular data of mitochondrial and SSR markers. These results reinforce the importance to couple morphological, ecological, and molecular data to have a broader perspective of biodiversity, especially in such an understudied environment as the marine realm. Following the results of this study and life-traits of *C. trifasciata*, we propose that the insular and mainland populations are currently experiencing a split, constituting distinct evolutionary lineages. As *C. trifasciata* is the only known representative of the genus in the NE Atlantic Ocean, further studies including more populations across the established distribution range of the species in the NE Atlantic Ocean, as well as from the putative populations in the Mediterranean Sea, might clarify questions regarding its current taxonomic classification.

### Population structure of *Cingula trifasciata* in the Azores Archipelago

**Fig. 8:**
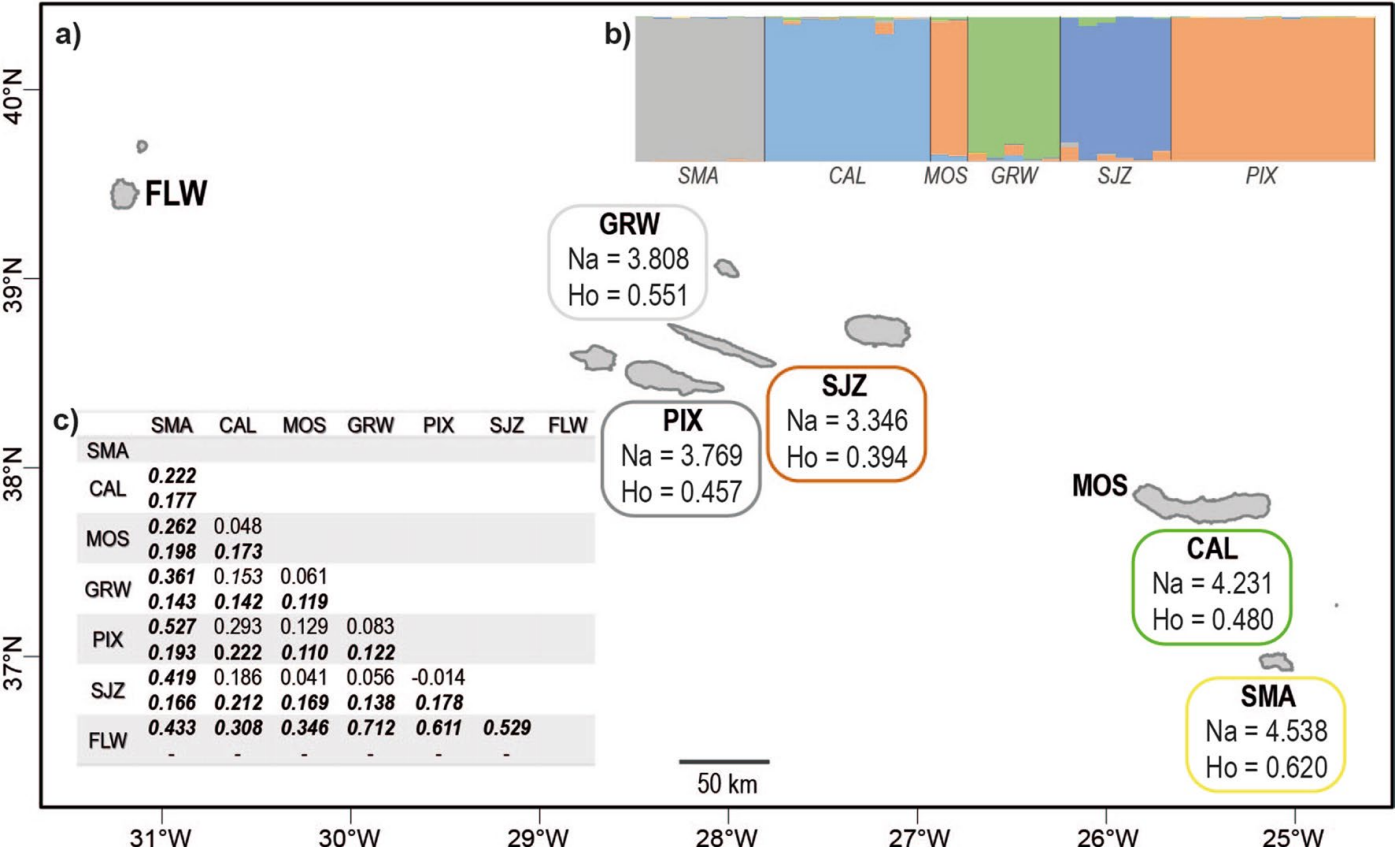
Summary of the relationships among Azorean populations of *Cingula trifasciata*. **a** Geographical map of the Azores Archipelago, with identification of the populations studied: SMA (Santa Maria), CAL (Caloura), MOS (Mosteiros), PIX (Pico), SJZ (São Jorge), GRW (Graciosa), and FLW (Flores). Coastline from the Portuguese Hydrographic Institute and bathymetry derived from GEBCO [46, 47]. The number of alleles (Na) and observed heterozygosity (Ho), based on the WAI dataset are presented for the populations with more than five individuals; **b** STRUCTURE v2.3.4 [89, 90] plot for the optimal K = 6; and **c** Pairwise FST values between populations of *C. trifasciata*; for each comparison, top values refer to the COI dataset, bottom values are based on the WAI dataset; bold italic indicates significant pairwiseF_ST_ values (p < 0.01)

The analyses of mitochondrial and SSR datasets provide insights at two different time scales, and both point to a strong genetic structure of *C. trifasciata* within the Azores Archipelago, suggesting low dispersal among islands. For Graciosa, Flores, and Mosteiros the discordant number of individuals analysed for both datasets can be explained by older samples or poorer preservation, causing their degradation and easier amplification of the COI. The main findings regarding the relationships among Azorean populations of *C. trifasciata* are summarized in a geographical map of the archipelago (Fig. 8).

Mitochondrial loci as COI are used for sequence analysis, indicated for the study of older divergence events and molecular differentiation of species. In their turn, SSR are a multilocus method in which differences are defined via allele frequency, making them especially useful to study spatial patterns of diversity at smaller time and geographic scales [109]. In that regard, the mitochondrial results suggest the occurrence of two haplogroups: 1) a star-like distribution mainly from the central group of the Azores (Pico, São Jorge, and Graciosa) suggest a recent differentiation in the region; 2) a linear branched haplogroup mainly from the eastern (Santa Maria and São Miguel) and western (Flores) groups, as a sign of stable, isolated populations with less frequent gene flow. Several explanations can be hypothesised for this observation such as the retention of ancestral polymorphisms, as Flores is separated from Santa Maria and São Miguel by more than 585 km of deep, unsuitable waters. Restrictions to gene flow of Flores’ populations had already been reported in the marine realm for *Patella candei* d'Orbigny, 1839 and *P. ulyssiponensis* Gmelin, 1791 [112]. Future fieldwork in more islands and localities in the Azores might allow more accurate inferences and reduce the number of unknown/unsampled mitochondrial haplotypes. Based on the WAI dataset, a stronger genetic structure is detected among populations. The clustering pattern in STRUCTURE roughly corresponds to a gradual East–West transition, except for the assignment of Graciosa and Mosteiros samples (cf. Figure 6). These deviations from the geographical pattern indicate a recent colonization from the Central group or the recent exchange of individuals due to unusual patterns of circulation between these island groups. Therefore, even though the Azorean islands are relatively isolated for long periods of times, occasional dispersal events of rafting and extreme weather might allow the exchange of individuals among distant populations [2, 48, 122].

Dispersal ability, habitat type, and oceanic circulation patterns within the archipelago, appear to be the main drivers of population structure in the Azores. The finding of strong genetic structure of *C. trifasciata* in the Azores is consistent with its np-larval development and habitat preferences of this microgastropod [51, 73–75, 77, 123, 124]. The lack of a free-swimming dispersal stage during the larval development of *C. trifasciata* likely hampers dispersal ability at early-life stages [6, 32, 33]. Considering the direct development strategy, most of the dispersal must be ensured by early juveniles and adults in occasional rafting in algal patches [2, 5, 125], as reported from other direct developers (e.g. *Batillaria cumingi* (Crosse, 1862) [124]). *C. trifasciata* gathers the common characteristics of rafters [126, 127], namely its minute size and presence among/close to algal patches in the intertidal. Rafting can be the process behind some inter-island dispersal events in the Azores Archipelago.

In the Azores, *C. trifasciata* and other microgastropod fauna are easily found in protected sites, under boulders in enclosed tide pools with low hydrodynamics and mesotidal regimes [e.g. Cerco da Caloura, Poça da Barra, and Fajã da Caldeira do Santo Cristo [128], Mosteiros]. *C. trifasciata* has been reported in similar habitats outside of the Azores (Lough Hyne, Ireland [26]). This rissoid has also been reported in Ceuta [34] and Praia de Lobos (Santa Maria Island) associated with gravel/boulders, which get uncovered during low tide but prone to wave action in bad weather conditions or high tide. In the absence of such conditions, they are found in lower numbers in algal substrates or among big boulders in protected intertidal areas of the Azorean islands, as reported for Flores and Graciosa islands [14]. The influence of habitat in the likelihood of dispersal events of juveniles and adults by rafting, thus affecting population structure, has been proposed to *Steromphala* spp. [74]. In our study system, one expects juveniles to drive genetic exchange in the gravel/boulder habitats, depending on the oceanographic features in the area. If the fauna is mainly associated with algal substrate, the chances of rafting in algae to distant localities are higher [2, 6]. The habitat type seems to play a major role at São Miguel Island, particularly at the enclosed locality of Caloura [14, 30, 34]. Migration events from and to Caloura seem to be rare, as the environment might hamper dispersal of juveniles from leaving the protected area and the low algal coverage reduces the chances of adult rafting. The surprisingly low connectivity between Caloura and Mosteiros, just 45 km apart, can be attributed to the geomorphological characteristics of the southern coast of São Miguel Island, which features long stretches of unsuitable sandy habitats for *C. trifasciata*, making gene flow virtually impossible between these populations. Mosteiros closely resembles populations from Graciosa and Pico (central group), located 215 to 225 km far, respectively. The sea-surface circulation regimes nearby the westernmost tip of São Miguel Island, where Mosteiros is located, might favour the frequent dispersal of individuals to or from the Central Group. Within the central islands, gene flow seems to be frequent and results point to recent haplotype differentiation. The distance separating the islands sampled in the central group (20 km Pico-São Jorge; 35 km São Jorge-Graciosa) is probably small enough to not constitute a barrier for the occasional migration and demographic expansion, supported by the low mitochondrial differentiation in levels comparable to other gastropods [65, 106].

The success of rafting episodes and maintenance of connectivity are influenced by the complex sea-surface circulation patterns and hydrological conditions around the Azores Archipelago [129–131]. Although surface spatial differences between islands are attributed to large-scale circulation in the NE Atlantic, patterns at fine scale close to island shores are far from well-understood but likely related to colder waters from upwelling or mixing processes [129]. Lagrangian transport pathways in the NE Atlantic Ocean are useful to understand potential rafting routes and major transport directions in the open ocean [130, 131]. These studies show the complexity of superficial (0–5 m) connectivity in the Azores area and among island groups, supporting our inferences regarding gene flow and exchange of individuals among populations: the western islands are the most isolated with lower changes of exchange with the remaining; high connectivity levels are expected within the Central Group; the Eastern Group is isolated to some extent but with some degree of connectivity with the Central Group [130, 131]. While the direction and circulation of the transport pathways within and among island groups await further studies, one cannot ignore the potential role of temporary disruptions of these circulation patterns in the episodic long-distance transports of individuals [48], often due to extreme weather events lashing the Azores. Future long-term studies regarding oceanographic circulation and extreme events in the NE Atlantic might provide the necessary data to better explain the genetic structuring of *C. trifasciata* and other np marine invertebrates in the Azores Archipelago.

## Conclusions

Combining mitochondrial and SSR datasets provide the opportunity to detect hidden patterns of cryptic diversity and to get a clearer perspective of gene flow among populations. Differentiation between *C. trifasciata* from the Azores and Vigo suggest the existence of potential cryptic diversity within the genus. Based on genetic data and life-history traits, these constitute distinct evolutionary lineages. Deep insights into spatial genetic structure of a microgastropod in the isolated Azores Archipelago were also reached with this study, providing crucial knowledge to better understand gene flow and connectivity among islands and considerably isolated populations. Several aspects seem to work in concert to shape the genetic structure of *C. trifasciata* in the Azores. Its np nature hinders dispersal during larval stage, but occasional exchange of individuals must occur through processes of rafting or by extreme weather events. Our results, especially the SSR dataset, reveal a preliminary analysis of these patterns in the remote islands of the Azores. A most comprehensive interpretation can only be achieved with further studies in related research areas, namely oceanography, but also on expanding molecular approaches to other marine invertebrates. Without a thorough understanding of fine-scale circulation patterns within the Azores, unexpected connections, as the one detected between Mosteiros and Graciosa, become difficult to justify. The application of the SSR-GBAS approach to other marine invertebrates might be the key to generate comparative datasets and to determine how widespread are the patterns inferred for *C. trifasciata*. How transversal is the complex interaction between the larval development type, ecological traits related to the habitat occupied, and the sea-surface circulation patterns in the population dynamics and genetic structure of marine invertebrates can be further addressed by expanding the taxa studied. Understanding differentiation levels and patterns of diversity at the regional scale might be a useful proxy for other intertidal invertebrate species, and to implement conservation and management strategies of the shore habitats in a way that connectivity in the remote Azores Archipelago will be preserved.

## Methods

With this work we aim to investigate the processes shaping the intraspecific genetic diversity and population dynamics of *C. trifasciata* in the NE Atlantic Ocean, with a special focus on its behaviour and factors shaping the genetic structure in the remote Azores Archipelago.

### Sampling and DNA isolation

*Cingula trifasciata* specimens from the Azores Archipelago (Portugal) and Vigo (Spain) were used in this study, either recently collected by the authors in intertidal habitats or retrieved from collections (Table 3). Fresh samples from Flores Island were obtained collected by integral algal scrapping with 20 × 20 cm squares. In other Azorean localities, numerous populations of *C. trifasciata* are sheltered under coastal boulder deposits in intertidal pools: Santa Maria, São Miguel, São Jorge, and Pico. All the recently collected specimens were stored in 96% ethanol and deposited in the Marine Molluscs Collection of the Department of Biology of the University of the Azores (DBUA). Permits for sampling were issued by the respective authorities in the Azores (Direção Reginal da Ciência e Tecnologia, Governo Regional dos Açores; AMP 2018/014, CCIP 24/2019/DRCT, CCIP 35/2019/DRCT). Samples from two localities in Graciosa island and from a NE Atlantic population in Vigo were loaned, respectively, from the DBUA and CIBIO-InBIO molluscs’ collections. A total of 75 samples were considered in this study.

**Table 3:** Samples of *Cingula trifasciata* used in this study

Island/Region	Locality	Habitat type	Coordinates (DD)	Collection voucher	#ind. (COI)	#ind. (SSR)
Santa Maria, Azores	Praia de Lobos	G/B	37.004, − 25.166	DBUA 1400	7	7
São Miguel, Azores	Caloura	G/B	37.71, − 25.51	DBUA 1474	11	9
	Mosteiros	G/B	37.891, − 25.825	DBUA 1194	11	2
São Jorge, Azores	Fajã da Caldeira de Santo Cristo	G/B	36.63, − 27.927	DBUA 1415	7	6
Pico, Azores	Poça da Barra	G/B	38.391, − 28.254	n.a./DBUA 1510	12	11
Graciosa, Azores	1-Poças de Santa Cruz	A	39.088^a^, − 27.997^a^	DBUA 1184	4	2
	2-Praia de São Mateus	A	39.087^a^, − 28.006^a^	DBUA 1186/1190	10	3
Flores, Azores	Poça da Salema	A	39.46, − 31.127	DBUA 1339	7	1
Galicia, Spain	Vigo	U	n.a	VIG4395-9	6	3

^a^Approximate coordinates; *n.a.* non-applicable; *G/B* Gravel/Boulder; *A* Algal mat; *U* Unknown; *DD* Decimal Degrees

Number of individuals studied for each population resorting to COI and SSR datasets are indicated

Due to its minute size, total genomic DNA (gDNA) was extracted from the entire animal, removed from the shell when possible, following the manufacturer’s instructions for the column-based commercial kit PureLink® Genomic DNA (Invitrogen™). gDNA was eluted in a volume of 40 μl and its quality assessed based on the absorbance ratios measured with Nanodrop®2000. An electrophoretic run in agarose gel 0.8% at 100 V for 50 min was performed to evaluate DNA integrity.

### Sequence data and genetic analyses

The fragment of the mitochondrial cytochrome oxidase subunit I (COI) were amplified in 25 μl volumes, containing 12.5 μl of QIAGEN Multiplex PCR Master Mix (Qiagen, CA, USA), 5 µL of each primer at a concentration of 2 μM, and 2.5 µL of gDNA. The amplification of COI was achieved with the primers LCO1490/HCO1490 [132] or jgLCO1490/jgHCO2198 [133]. The following cycling profile was applied to both markers: 95 °C for 15 min; 35 cycles of 95 °C for 30 s, 50/55 °C for 1 min, 72 °C for 30 s; 72 °C for 10 min. PCR products were checked by electrophoresis. Purification of the PCR products and bi-directional Sanger sequencing were realized by a commercial facility (Genewiz, Leipzig, Germany) using the same primers as for PCR.

Geneious 8.1.9 [84] was used for manual check of potential misreads in the generated chromatograms. The reviewed mitochondrial coding COI sequences were inspected for the existence of stop codons and putative pseudogenes by translating into amino acids with ExPASy Translate Tool [134]. All the sequences generated during this study were deposited at GenBank [86], under the accession numbers MW518062-117 and MW518858-63. Additional sequences of *C. trifasciata* from the Azores, publicly available at GenBank [86], were included in the COI dataset: MG652395-MG562406. The COI dataset was aligned with Clustal Omega algorithm via Web Services by EMBL-EBI [135] and the 75 sequences were reduced to 44 haplotypes. Raw (p) distances among haplotypes of *C. trifasciata* were calculated in MEGA v7 [136] to estimate evolutionary divergence and sequence diversity. Frequency, distribution, and genetic structure between the haplotypes in the populations was further examined at a statistical parsimony haplotype network at the 95% connection limit, generated with the software TCS v1.21 [81]. The output was rendered using tcsBU web-based program [82], allowing to overlap the genetic structure retrieved by TCS with the geographical structure of the populations studied. A UPGMA tree of *C. trifasciata* haplotypes [83] was obtained with Geneious 8.1.9 [84], and bootstraps calculated with 1,000 replicates. The evolutionary divergence levels within Rissoidae family were further evaluated with a maximum-likelihood analysis conducted in RAxML v8.2.7 [85], under the GTR model of substitutions and based on 18 COI sequences of different rissoid species retrieved from GenBank [86] (cf. Additional file 1: Table S5 for details) and *C. trifasciata* from the Azores and Vigo. Pairwise F_ST_ estimates with COI haplotypes of *C. trifasciata* and Rissoidae species were performed in Arlequin v3.5 [137], with statistical significance tested by 1023 permutations and considered for p-values below 0.01.

### SSR marker discovery

Two low-coverage Illumina MiSeq runs, from two *C. trifasciata* individuals (PSM4350 from Graciosa and FSC5131 from São Jorge), were conducted for marker development and raw reads can be accessed through the BioProject PRJNA702169, at GenBank [86]. Library preparation and sequencing using shot-gun genomic libraries without enrichment on the Illumina MiSeqs paired-end (PE) 300 bp were performed at the Genomics Service Unit, Ludwig-Maximilian University Munich, Germany. The resulting reads, after quality check with FastQC software[138], were processed by Trimmomatic v0.39 [139] to trim adapters and low-quality regions (Phred score > 20) in forward and reverse reads, which were then merged using Usearch v11 [140]. Merged reads were used as input for the identification of sequences containing microsatellite (SSR) motifs with the SSR_pipeline’s script SSR_search.py [141]. For the SSR search, the following parameters of quality control were defined to ensure that the sequence contains: 1) a minimum of 30 bp flanking regions on both sides of the motif; 2) a minimum of five repeats for penta- and tetranucleotides; 3) a minimum of seven repeats for trinucleotides; 4) a minimum of nine repeats for dinucleotides. These less stringent parameters allowed the extraction of a considerable number of SSR motif containing sequences, which were manually checked to remove sequences containing either interrupted motifs, more than one repetitive motif, and long mononuclear stretches (> 6 bp). If this filtering step resulted in a low final number of usable reads, sequences containing mononucleotide repeats were maintained.

Primer3 [142] as implemented in Geneious 8.1.9 [84] was chosen to design primers, as a batch under manual control. The following parameters were set for the primer design: length of 19 to 22 bp, optimal melting temperature of 55 °C, GC content between 20 and 80% and optimal as 50%, and amplification product size between 300 and 450 bp. Only primers producing amplicons comprising the complete SSR repetitive motif in the first or last 300 bp were selected, ensuring its coverage by one of MiSeq’s paired reads. This is crucial to avoid problems in the overlap of the paired reads during the merging step of the bioinformatics pipeline [91, 92]. We added recognition sequences corresponding to the Illumina adapter to the selected primer pairs: part of the P5 motif (TCTTTCCCTACACGACGCTCTTCCGATCT) elongated the forward primer, whereas part of the P7 motif (CTGGAGTTCAGACGTGTGCTCTTCCGATCT) was added to the reverse primer. These recognition sequences serve as linkers for a second index PCR using primers containing the eight bp indexes and TrueSeq adapters (P5: AATGATACGGCGACCACCGAGATCTACAC [Index] ACACTCTTTCCCTACACGACG; and P7: CAAGCAGAAGACGGCATACGAGAT [Index] TGACTGGAGTTCAGACGTGT) [91, 92]. The primers designed from the initial two Illumina MiSeq runs were individually tested using gDNA for two specimens of *C. trifasciata.* PCR reactions were conducted for a final volume of 10 µL: 5 µL of QIAGEN Multiplex PCR Master Mix (Qiagen, CA, USA), 1 µL of each primer at 1 µM, 1 µl of diluted gDNA in a 1:3 proportion, and water. The cycling profile was applied as follows: 95 °C for 15 min; 30 cycles of 95 °C for 30 s, 55 °C for 1 min, and 72 °C for 1 min; and a final extension at 72 °C for 10 min. The PCR results were visualized after an electrophoretic run in 1.5% agarose gel and 80 V. Primers that successfully generated amplicons of the expected size were combined in three mixes of eight primer pairs and one mix of seven primer pairs, each with a final concentration of 1 µM (Additional file 1: Table S1).

### Multiplex PCR and Illumina sequencing

Multiplex amplification was achieved with PCR reactions of 5 µL, containing 2.5 µL of QIAGEN Multiplex PCR Master Mix (Qiagen, CA, USA), 0.5 µL of each primer mix, and 2 µL of gDNA diluted in 1:3 proportion. Cycling conditions were the same as used in the single PCR to test the primers individually. For each sample, and following Curto et al.’s (2019) protocol, equal volumes of PCR products from different primers mixes were pooled into a final volume of 6 µL. PCR clean-up, aiming the removal of unused primers and primer-dimer products, was conducted using the magnetic bead technology offered by the Agencourt AMPure XP PCR Purification kit (Beckman Coulter Inc., Bree, CA, USA), applying some modifications to the standard protocol. The total volume of pooled PCR product (6 µL) was mixed with 4.3 µL of AMPure XP beads, followed by a 5 min incubation period at room temperature. The beads, to which DNA bound after the first step, were captured by an inverted magnetic bead extraction device, VP407‐AM‐N (V&P Scientific, INC.), and afterwards washed twice for 45 s in 200 µL of 80% ethanol. The beads were then dried at room temperature for 5 min and the DNA finally eluted in 17 µL of elution buffer (10 mM Tris–HCl, pH 8.3) at 65 °C.

Once purified, the multiplex PCR product underwent a second PCR, whereby designated as index-PCR, for the assignment of indexes to each sample. A unique combination of forward and reverse indexes was carefully chosen, so that each sample can be unambiguously identified after the MiSeq Run. A 10 µL PCR reaction was performed, comprising 5 µL of QIAGEN Multiplex PCR Master Mix (Qiagen, CA, USA), 1 µL of each index primer at 1 µM, and 1 µL of pooled purified PCR product. The cycling conditions were applied as follows: 95 °C for 15 min; 10 cycles of 95 °C for 30 s, 58 °C for 60 s, and 72 °C for 60 s; 72 °C for 5 min. The index-PCR product, from 5’ to 3’, entails: P5 motif for flow cell hybridization, index 1 with a length of 8 bp, P5 sequencing primer, specific forward primer, target DNA for sequence, specific reverse primer, P7 sequencing primer, 8 bp long index 2, P7 motif for flow cell hybridization. After visualizing the index-PCR products in 1.5% agarose gel stained with GelRed (Biotium), all the samples were pooled in equal volumes of 2 µL. The Illumina MiSeq run for PE 300 bp sequencing at the Genomics Service Unit at Ludwig Maximillian Universität, München, Germany, using the pooled index-PCR product as input, produced sequences to be analysed with a genotyping by amplicon sequencing (GBAS) protocol.

### SSR data analysis and SSR-GBAS genotyping

Raw FASTQ sequence data (reads R1 and R2), automatically extracted by the MiSeq equipment based on the index combinations and each corresponding to a different sample, were downloaded from Illumina BaseSpace. These Illumina reads, containing all sequences per index, underwent the quality control and merging procedures resorting to FastQC, Trimmomatic v0.39, Usearch v11 (cf. “SSR discovery” section). Genotyping and analysis of each sample for all the SSR loci was performed with the SSR_GBS_pipeline scripts available at GitHub [92, 143]. Script 1 (primer_demultiplex.py) allowed to demultiplex merged fastq files by identifying the primers on both sides of the merged reads and sort them by locus. Script 2 (CountLengths.sh) calculated the number of occurrences of each sequence length, excluding sequences below the 250 bp threshold. With Script 3, potential alleles and histograms were plotted according to sequence length, setting a minimum of 10 reads to define a valid genotype. The stutter effect in the defined alleles is also assessed by Script 3, as described previously [91, 92]. A codominant matrix in.csv format, based on the allele length information of each sample is generated and used as input for the downstream recovery of sequence alleles, which considers SNP variation within alleles of the same size along with the sequence length variation determined at this point. Sequence_Allele_Call.py (Script 4) constitutes the second part of the SSR_GBS_pipeline.py, for the extraction of the corresponding reads and definition of consensus sequence for each length-based allele. It also allows the detection of possible SNP variation and calls alleles based on sequence information. This information set is called in the following WAI.

### Population structure analyses

Descriptive population genetics and marker variability analyses, as well as evaluation of genetic structure patterns, were performed on the codominant WAI dataset of *Cingula trifasciata*. The two populations of Graciosa island were analysed together, as no major differences were found between them. Genetic diversity indices, null alleles and deviations to Hardy–Weinberg Equilibium (HWE) in the WAI dataset were estimated only for populations with five or more individuals analysed – Santa Maria, Caloura, Graciosa, Pico, and São Jorge. FreeNA [144] was used to estimate the frequency of null alleles per marker and population, using the AL dataset as required by the software. GenAlEx v6.5 [87, 88] allowed to check the number of alleles per marker and allelic patterns per population – number of alleles (Na), number of private (Npa) and effective (Ne) alleles, observed (*Ho*) and expected (*He*) heterozygosity. Although excluded from the inference of genetic diversity, monomorphic markers were kept in the dataset for downstream analyses, as they are informative [145]. Tests for deviations to HWE and Wright’s Fixation index (F_IS_) were performed by marker and population resorting to GenAlEx. The remaining analyses based on the WAI dataset included information of all individuals studied, belonging to the seven populations sampled. The software Cervus v3.0.7 [146] was used to estimate the overall polymorphism information content (PIC) of each marker. Genetic structure patterns in the dataset were inferred in a Principal Coordinate Analysis (PCoA), as implemented in GenAlex v6.5 [87, 88], and STRUCTURE v2.3.4 [89, 90] with the complete dataset, allowing to assess the informativeness of the developed SSR markers. PCoA, performed with the complete dataset and excluding Vigo to clarify the position of the Azorean populations, evaluates genetic structure among populations without assumptions of HWE and based on absolute genetic distances between individuals. With the number of clusters (K) varying between 2 and 10, STRUCTURE ran for 10 independent replicates for 500,000 generations, following a burn-in period of 100,000 and maintaining the default settings for the admixture model and correlated allele frequencies [147]. The online program Structure Harvester [148] was used to validate multiple K-values for optimal detection of genetic structure, according to the Delta-K method and inferring the K that best suits the data from hundreds of iterations. The results from STRUCTURE across the K-values were summarized and graphically displayed resorting to the online pipeline CLUMPAK [149]. A hierarchical analysis of molecular variance (AMOVA [150]), as implemented in GenAlEx v6.5 [87, 88], was performed to evaluate the differentiation between populations and regions, with a simultaneous estimate of pairwise population F_ST_ values.

## Supplementary Information

**Additional file 1: Table S1.** Complete list of all SSR primers designed in this study. Includes sequence of forward and reverse primers (5’-3’), the repetition motif, and number of repeats in the original sequence used for the primer design, the primer mix in which it was included for the Multiplex PCR, and allele length range (bp). **Table S2.** Estimates of evolutionary divergence (raw p-distances) between COI haplotypes of *Cingula trifasciata*. The analysis, performed on MEGA v7 [1], considered 44 haplotypes and 658 bp after excluding positions containing gaps and/or missing data for each sequence pair analysed. Populations of the sequences collapsed within each haplotype is referred in the “Pops” column, as follows: SMA-Santa Maria, CAL-Caloura, MOS-Mosteiros, GRW-Graciosa, PIX-Pico, FSC-São Jorge, FLW-Flores, VIG-Vigo. **Table S3.** Pairwise FST values between populations of *Cingula trifasciata*, based on the COI dataset. Table S4—Pairwise FST values between populations of *Cingula trifasciata*, based on the complete WAI dataset. Table S5 – Estimates of evolutionary divergence (raw p-distances) between COI sequences of several Rissoidae species. The analysis, performed on MEGA v7 [1], considered 18 sequences and 658 bp after excluding positions containing gaps and/or missing data for each sequence pair analysed. Sequences retrieved from GenBank [2], accession Number (AN) and species identification are provided in the table. Highlighted in bold is the divergence level between the recognized species *Alvania formicarum* and *A. mediolittoralis*, and between *Cingula trifasciata* from the Azores and Vigo. **Figure S1.** Genetics diversity of the SSR dataset in study. a) Variability measures estimated for each of the 26 SSR loci in study in complete whole amplicon information (WAI) dataset: number of alleles (Na) and polymorphic information content (PIC); b) Genetic diversity patterns across the five populations of *Cingula trifasciata* with more than five individuals: number of alleles (Na), number of different alleles with frequency over 5%, number of effective alleles (Ne), number of private alleles (Npa), expected (He) and observed (Ho) heterozygosity. **Fig. S2.** Genetic structure analysis of the complete WAI dataset of *Cingula trifasciata.* Inferred with STRUCTURE v2.3.4 [5, 6], reporting the results from K = 7 to K = 10.

## Data Availability

All the mitochondrial COI sequences of *Cingula trifasciata* generated in this study have been deposited at GenBank database, under the accession numbers MW518062-117 and MW518858-63. Raw reads from the low-coverage whole-genome sequencing library used for marker development and WAI dataset can be accessed through the BioProject PRJNA702169. The WAI allele sequences were submitted to GenBank database, under the accession numbers MW623085-363. Public access to the databases is open.

## Abbreviations

AMOVA: Hierarchical analysis of Molecular Variance
COI: Cytochrome oxidase subunit I
DBUA: Department of Biology of the University of the Azores
GBAS: Genotyping by Amplicon Sequencing
gDNA: Genomic DNA
HWE: Hardy–Weinberg Equilibrium
Ma: Million years
NGS: Next-generation sequencing
np-: Non-planktotrophic
PCoA: Principal Coordinates Analysis
PCR: Polymerase Chain Reaction
SNP: Single Nucleotide Polymorphism
SSR: Simple Sequencing Repeats/ microsatellites
SSR-GBAS: Simple Sequencing Repeats—Genotyping by Amplicon Sequencing
WAI: Whole Amplicon Sequencing
