# Supplementary material for: Dispersal ability, habitat characteristics, and sea-surface circulation shape population structure of Cingula trifasciata (Gastropoda: Rissoidae) in the remote Azores Archipelago
Source: BMC Ecol Evol. 2021 Jun 22;21:128. doi: 10.1186/s12862-021-01862-1 (PMC8218459; doi:10.1186/s12862-021-01862-1)
Supplement: Supplementary file 1 — Additional file 1: Table S1. Complete list of all SSR primers designed in this study. Includes sequence of forward and reverse primers (5’-3’), the repetition motif, and number of repeats in the original sequence used for the primer design, the primer mix in which it was included for the Multiplex PCR, and allele length range (bp). Table S2. Estimates of evolutionary divergence (raw p-distances) between COI haplotypes of Cingula trifasciata. The analysis, performed on MEGA v7 [1], considered 44 haplotypes and 658 bp after excluding positions containing gaps and/or missing data for each sequence pair analysed. Populations of the sequences collapsed within each haplotype is referred in the “Pops” column, as follows: SMA-Santa Maria, CAL-Caloura, MOS-Mosteiros, GRW-Graciosa, PIX-Pico, FSC-São Jorge, FLW-Flores, VIG-Vigo. Table S3. Pairwise FST values between populations of Cingula trifasciata, based on the COI dataset. Table S4—Pairwise FST values between populations of Cingula trifasciata, based on the complete WAI dataset. Table S5 – Estimates of evolutionary divergence (raw p-distances) between COI sequences of several Rissoidae species. The analysis, performed on MEGA v7 [1], considered 18 sequences and 658 bp after excluding positions containing gaps and/or missing data for each sequence pair analysed. Sequences retrieved from GenBank [2], accession Number (AN) and species identification are provided in the table. Highlighted in bold is the divergence level between the recognized species Alvania formicarum and A. mediolittoralis, and between Cingula trifasciata from the Azores and Vigo. Figure S1. Genetics diversity of the SSR dataset in study. a) Variability measures estimated for each of the 26 SSR loci in study in complete whole amplicon information (WAI) dataset: number of alleles (Na) and polymorphic information content (PIC); b) Genetic diversity patterns across the five populations of Cingula trifasciata with more than five individuals: number of alleles (N [file 12862_2021_1862_MOESM1_ESM.docx]

**Additional Files**

**Using SSR-GBAS to infer the population structure of *Cingula trifasciata* (Gastropoda: Rissoidae) in the Azores Archipelago (NE Atlantic Ocean)**

Baptista L^1,2,3,4^, Meimberg H^1^, Ávila SP^2,3,4,5^, Santos AM^3,6^, Curto M^1,7^

^1^ Institute for Integrative Nature Conservation Research, University of Natural Resources and Life Sciences (BOKU), Vienna, Austria; ^2^ CIBIO, Centro de Investigação em Biodiversidade e Recursos Genéticos, InBIO Laboratório Associado, Pólo dos Açores, 9501-801 Ponta Delgada, Azores, Portugal; ^3^ MPB-Marine Palaeontology and Biogeography lab, University of the Azores, Rua da Mãe de Deus, 9501-801 Ponta Delgada, Azores, Portugal; ^4^ Faculdade de Ciências da Universidade do Porto, Rua do Campo Alegre 1021/1055, 4169-007 Porto, Portugal; ^5^ Departamento de Biologia, Faculdade de Ciências e Tecnologia, Universidade dos Açores, 9501-801 Ponta Delgada, Azores, Portugal; ^6^ CIBIO, Centro de Investigação em Biodiversidade e Recursos Genéticos, InBIO Laboratório Associado, Universidade do Porto, Campus de Vairão, Rua Padre Armando Quintas, no. 7, 4485-661 Vairão, Portugal; ^7^ MARE, Marine and Environmental Sciences Centre, Faculdade de Ciências, Universidade de Lisboa, Campo Grande, Lisboa, 1749-016 Portugal

**Table S1** *-* **Complete list of all SSR primers designed in this study**. Includes sequence of forward and reverse primers (5’-3’), the repetition motif, and number of repeats in the original sequence used for the primer design, the primer mix in which it was included for the Multiplex PCR, and allele length range (bp).

| Primer/ Loci | Forward (5’-3’) | Reverse (5’-3’) | Repetition motif | Mix | Allele length range (bp) |
| --- | --- | --- | --- | --- | --- |
| CT1_ATGA | CCCTACTTTCAGCAGGAGAA | TGCGATCCTTATACCGCTTT | (TGTT)5 | 4 | Failed (>60 % missing data) |
| CT3_GAATA | CCGGACAGTGTAATTATAGCA | ACGGCGGTTGAATTGACTA | (GAATA)7 | 3 | Failed (>60 % missing data) |
| CT5_TGAA | CCTGGTTAATTTTCAGCGCA | GGTGGCGGTATATTCAGCTA | (TGAA)6 | 3 |  |
| CT6_TTGT | TAGTGTTCGCTTGCTTGTTG | TAGTGTTCGCTTGCTTGTTG | (TTGT)5 | 1 | Failed (non-specific marker) |
| CT10_GTTT | TCTATCGGTGATGTGTTGCA | GGACTAGACAGAATCCGAGC | (GTTT)5 | 2 | Failed (>60 % missing data) |
| CT11_GTTT | GATCTGCTCAGTGAACATGC | GGGTGAGACAGGTTGAGAAA | (GTTT)5 | 4 |  |
| CT13_GTTG | ATACTGGGATGCACAGGAAC | ACGGAATTAAAACAAGCGCA | (GTTG)5 | 2 |  |
| CT14_CAGA | AGAAACGAGCTCATTCTTGT | CGTGGCCTTGCTTTTCTAAA | (CAGA)9 | 4 |  |
| CT15_TTTG | AAGCTCCATATCAACAGCCA | TCCTGTTGTCGAGAAGATGG | (TTTG)5 | 1 |  |
| CT16_TTTG | TGACACGAGTCTGACGTAAG | CCCCACTTATACTGTGCCTT | (TTTG)5 | 3 |  |
| CT17_TTTG | AAGCGTTCTAGGACTGAACC | TTCTGAACATCGACACCACA | (TTTG)5 | 2 | Failed (non-specific marker) |
| CT18_AAAG | GACAGAAGGTCCAGACATTTT | TGACACGAGTCTGACGTAAG | (AAAG)5 | 3 |  |
| CT19_GACA | ACTGGCAAAGTCCTTCTCAA | CCCAATCATCACGGTCAAAC | (GACA)6 | 4 |  |
| CT21_CAAA | GAACGAAAGGGAAACGGATG | GAGACGGGTTCTCTGACATT | (CAAA)5 | 1 |  |
| CT22_ACAG | AACTCCATCCGCCATGTAAT | TTGCTCACATTTCACTGTGC | (ACAG)7 | 2 |  |
| CT23_TTTA | AACTTTTGATCTTGCTGCCG | TCTGAATTCGGGGTTTGTCA | (TTTA)5 | 3 |  |
| CT24_CAAA | CAATGGTGCTAGCTTTCTGC | AGGCTTTGGATCTGGATCTG | (CAAA)6 | 2 |  |
| CT26_TGTT | CCTCACAGGCTCAAGATACA | GCGACAGAACAACGTTGTAA | (TGTT)5 | 4 |  |
| CT28_CTGT | ATTAACAGCTGGGTGGACC | TGAGCATAAGTGACCTGCAT | (CTGT)5 | 2 |  |
| CT29_AAAT | CCCAGAAAGAAAACCTTCACC | GCAAGCAACCTGTACTATGC | (AAAT)6 | 1 |  |
| CT30_TGTC | TGATCAGATGCCCACATTCA | GCGCACTTTAAGTTGAGAGA | (TGTC)5 | 2 |  |
| CT31_AATA | GGGGTTCATTCAGTGTTTACA | TGACATCCACTGAACAGGAG | (AATA)5 | 1 |  |
| CT32_TTTG | GGCGAGCTTTAAGTGACATC | ACATTCAGCATTCAGACCAA | (TTTG)6 | 4 |  |
| CT33_CATT | CTCCATGCAAAATTCCCAGG | CCACCCTCGTCCATTTAGAA | (CATT)5 | 3 |  |
| CT34_ACCA | TCTTGGGTTTGATGTGTGGA | ATGGTTAGGTGCAAGGGATC | (ACCA)5 | 1 |  |
| CT35_TTTG | GTGCCACTTTAAGAAACGCT | CGAAATTTGGAATCCGATACG | (TTTG)12 | 1 |  |
| CT37_AAAT | ACAGCCACAAGTTTGTCAAC | GTCCTTCTGTCTCTGCCTG | (AAAT)5 | 2 |  |
| CT38_TCAT | GTCCTTTACACAGCAAAGCT | TAGGATCGCCATCGGAGTT | (TCAT)8 | 3 |  |
| CT39_CATT | ATACTTCAGGCCGGAAAGAG | CTAAGCCTCAGCAGAGACTG | (CATT)6 | 1 |  |
| CT40_ATCC | GAGAACGAACTCTCAGCTACT | CTTCAAAGCACAGTGTAGGG | (ATCC)5 | 3 |  |
| CT42_TAAA | GCCAGTCCTCATACGAACAT | CTGCGTCTCAGATGTTTCAC | (TAAA)5 | 4 |  |
|  |  |  |  |  |  |
| CT2_TGTT | TGGCGCAATGAATAGAATGTC | AAAGAGGGCGCTATGTACTC | (TGTT)5 | - | Failed in single PCR test |
| CT4_TAGAA | TGCTTTTCATCAGTATCGTCA | TGATGCATTTGTGATCATTGA | (TAGAA)5 | - | Failed in single PCR test |
| CT7_TTATG | CCCCTTAAACGATTTCTGAGA | CTGGTAGGGAGGTTTACGAA | (TTATG)6 | - | Failed in single PCR test |
| CT8_AACAT | CTGCAGTGTCAAGTATGCAG | CGGTGGCAAGTTTCTGTATC | (AACAT)5 | - | Failed in single PCR test |
| CT9_GTCAA | TGGATACATCACGAGATCGG | TCACCTGTTGCACAAATCAT | (GTCAA)5 | - | Failed in single PCR test |
| CT12_GTTG | ATGGGTTACAAGATACGCGT | AGTGCACATTCAGTCCATCA | (GTTG)7 | - | Failed in single PCR test |
| CT20_ATAC | ACATCTGTCGTTCGTCTTCA | ACCGTTACCTCTCAATAGACT | (ATAC)5 | - | Failed in single PCR test |
| CT25_AAGA | GCCAGATGTATGTTTTCCCG | GTTGGGATTCAGGTCAGCTA | (AAGA)6 | - | Failed in single PCR test |
| CT27_CATT | GTGATGGAAGTACTCATGCG | TGGAATGGAAAGAGACGGAT | (CATT)10 | - | Failed in single PCR test |
| CT36_AGAC | GTAGTGTGCATGCCTAAGTG | CACAATTGTTTATGCCTTGGT | (AGAC)7 | - | Failed in single PCR test |
| CT41_TCAA | GCAAGGAGAGTGGAGGTTAC | ACGGAGTGATTATGGAAGGG | (TCAA)6 | - | Failed in single PCR test |

**Table S2 - Estimates of evolutionary divergence (raw p-distances) between COI haplotypes of *Cingula trifasciata*.** The analysis, performed on MEGA v7 [1], considered 44 haplotypes and 658 bp after excluding positions containing gaps and/or missing data for each sequence pair analysed. Populations of the sequences collapsed within each haplotype is referred in the “Pops” column, as follows: SMA-Santa Maria, CAL-Caloura, MOS-Mosteiros, GRW-Graciosa, PIX-Pico, FSC-São Jorge, FLW-Flores, VIG-Vigo.

| **Pops** | **Hap** | 1 | 2 | 3 | 4 | 5 | 6 | 7 | 8 | 9 | 10 | 11 | 12 | 13 | 14 | 15 | 16 | 17 | 18 | 19 | 20 | 21 | 22 | 23 | 24 | 25 | 26 | 27 | 28 | 29 | 30 | 31 | 32 | 33 | 34 | 35 | 36 | 37 | 38 | 39 | 40 | 41 | 42 | 43 |
| --- | --- | --- | --- | --- | --- | --- | --- | --- | --- | --- | --- | --- | --- | --- | --- | --- | --- | --- | --- | --- | --- | --- | --- | --- | --- | --- | --- | --- | --- | --- | --- | --- | --- | --- | --- | --- | --- | --- | --- | --- | --- | --- | --- | --- |
| SMA | **1** |  |  |  |  |  |  |  |  |  |  |  |  |  |  |  |  |  |  |  |  |  |  |  |  |  |  |  |  |  |  |  |  |  |  |  |  |  |  |  |  |  |  |  |
| SMA | **2** | 0.011 |  |  |  |  |  |  |  |  |  |  |  |  |  |  |  |  |  |  |  |  |  |  |  |  |  |  |  |  |  |  |  |  |  |  |  |  |  |  |  |  |  |  |
| SMA | **3** | 0.002 | 0.009 |  |  |  |  |  |  |  |  |  |  |  |  |  |  |  |  |  |  |  |  |  |  |  |  |  |  |  |  |  |  |  |  |  |  |  |  |  |  |  |  |  |
| SMA | **4** | 0.021 | 0.014 | 0.02 |  |  |  |  |  |  |  |  |  |  |  |  |  |  |  |  |  |  |  |  |  |  |  |  |  |  |  |  |  |  |  |  |  |  |  |  |  |  |  |  |
| SMA | **5** | 0.011 | 0.003 | 0.009 | 0.011 |  |  |  |  |  |  |  |  |  |  |  |  |  |  |  |  |  |  |  |  |  |  |  |  |  |  |  |  |  |  |  |  |  |  |  |  |  |  |  |
| SMA | **6** | 0.018 | 0.011 | 0.017 | 0.011 | 0.011 |  |  |  |  |  |  |  |  |  |  |  |  |  |  |  |  |  |  |  |  |  |  |  |  |  |  |  |  |  |  |  |  |  |  |  |  |  |  |
| CAL | **7** | 0.014 | 0.006 | 0.012 | 0.011 | 0.006 | 0.009 |  |  |  |  |  |  |  |  |  |  |  |  |  |  |  |  |  |  |  |  |  |  |  |  |  |  |  |  |  |  |  |  |  |  |  |  |  |
| CAL | **8** | 0.017 | 0.009 | 0.015 | 0.014 | 0.009 | 0.012 | 0.006 |  |  |  |  |  |  |  |  |  |  |  |  |  |  |  |  |  |  |  |  |  |  |  |  |  |  |  |  |  |  |  |  |  |  |  |  |
| CAL | **9** | 0.015 | 0.008 | 0.014 | 0.012 | 0.008 | 0.011 | 0.005 | 0.008 |  |  |  |  |  |  |  |  |  |  |  |  |  |  |  |  |  |  |  |  |  |  |  |  |  |  |  |  |  |  |  |  |  |  |  |
| CAL | **10** | 0.012 | 0.008 | 0.014 | 0.012 | 0.008 | 0.011 | 0.005 | 0.008 | 0.006 |  |  |  |  |  |  |  |  |  |  |  |  |  |  |  |  |  |  |  |  |  |  |  |  |  |  |  |  |  |  |  |  |  |  |
| CAL | **11** | 0.021 | 0.014 | 0.02 | 0.003 | 0.014 | 0.011 | 0.011 | 0.014 | 0.012 | 0.012 |  |  |  |  |  |  |  |  |  |  |  |  |  |  |  |  |  |  |  |  |  |  |  |  |  |  |  |  |  |  |  |  |  |
| CAL | **12** | 0.023 | 0.015 | 0.021 | 0.008 | 0.015 | 0.012 | 0.012 | 0.015 | 0.014 | 0.014 | 0.008 |  |  |  |  |  |  |  |  |  |  |  |  |  |  |  |  |  |  |  |  |  |  |  |  |  |  |  |  |  |  |  |  |
| CAL | **13** | 0.014 | 0.009 | 0.015 | 0.014 | 0.009 | 0.012 | 0.006 | 0.009 | 0.008 | 0.005 | 0.014 | 0.015 |  |  |  |  |  |  |  |  |  |  |  |  |  |  |  |  |  |  |  |  |  |  |  |  |  |  |  |  |  |  |  |
| MOS, GRW | **14** | 0.021 | 0.014 | 0.02 | 0.003 | 0.014 | 0.011 | 0.008 | 0.014 | 0.012 | 0.012 | 0.003 | 0.008 | 0.014 |  |  |  |  |  |  |  |  |  |  |  |  |  |  |  |  |  |  |  |  |  |  |  |  |  |  |  |  |  |  |
| MOS | **15** | 0.018 | 0.011 | 0.017 | 0.015 | 0.011 | 0.012 | 0.008 | 0.008 | 0.009 | 0.006 | 0.015 | 0.017 | 0.011 | 0.015 |  |  |  |  |  |  |  |  |  |  |  |  |  |  |  |  |  |  |  |  |  |  |  |  |  |  |  |  |  |
| MOS, FSC, PIX, GRW | **16** | 0.02 | 0.012 | 0.018 | 0.002 | 0.012 | 0.009 | 0.009 | 0.012 | 0.011 | 0.011 | 0.002 | 0.006 | 0.012 | 0.002 | 0.014 |  |  |  |  |  |  |  |  |  |  |  |  |  |  |  |  |  |  |  |  |  |  |  |  |  |  |  |  |
| MOS | **17** | 0.026 | 0.018 | 0.024 | 0.008 | 0.018 | 0.015 | 0.015 | 0.018 | 0.017 | 0.017 | 0.008 | 0.012 | 0.018 | 0.008 | 0.02 | 0.006 |  |  |  |  |  |  |  |  |  |  |  |  |  |  |  |  |  |  |  |  |  |  |  |  |  |  |  |
| MOS | **18** | 0.02 | 0.012 | 0.018 | 0.017 | 0.012 | 0.014 | 0.008 | 0.009 | 0.011 | 0.008 | 0.017 | 0.018 | 0.012 | 0.017 | 0.002 | 0.015 | 0.021 |  |  |  |  |  |  |  |  |  |  |  |  |  |  |  |  |  |  |  |  |  |  |  |  |  |  |
| MOS | **19** | 0.02 | 0.012 | 0.018 | 0.008 | 0.012 | 0.009 | 0.008 | 0.012 | 0.011 | 0.011 | 0.008 | 0.009 | 0.012 | 0.008 | 0.014 | 0.006 | 0.012 | 0.015 |  |  |  |  |  |  |  |  |  |  |  |  |  |  |  |  |  |  |  |  |  |  |  |  |  |
| MOS | **20** | 0.021 | 0.014 | 0.02 | 0.003 | 0.014 | 0.011 | 0.009 | 0.014 | 0.012 | 0.012 | 0.003 | 0.008 | 0.014 | 0.003 | 0.015 | 0.002 | 0.008 | 0.017 | 0.005 |  |  |  |  |  |  |  |  |  |  |  |  |  |  |  |  |  |  |  |  |  |  |  |  |
| MOS | **21** | 0.011 | 0.003 | 0.009 | 0.014 | 0.003 | 0.011 | 0.006 | 0.009 | 0.008 | 0.008 | 0.014 | 0.015 | 0.009 | 0.014 | 0.008 | 0.012 | 0.018 | 0.009 | 0.012 | 0.014 |  |  |  |  |  |  |  |  |  |  |  |  |  |  |  |  |  |  |  |  |  |  |  |
| PIX | **22** | 0.024 | 0.017 | 0.023 | 0.006 | 0.017 | 0.014 | 0.014 | 0.017 | 0.015 | 0.015 | 0.006 | 0.011 | 0.017 | 0.006 | 0.018 | 0.005 | 0.011 | 0.02 | 0.008 | 0.006 | 0.017 |  |  |  |  |  |  |  |  |  |  |  |  |  |  |  |  |  |  |  |  |  |  |
| PIX | **23** | 0.021 | 0.014 | 0.02 | 0.003 | 0.014 | 0.011 | 0.011 | 0.014 | 0.012 | 0.012 | 0.003 | 0.008 | 0.014 | 0.003 | 0.015 | 0.002 | 0.008 | 0.017 | 0.008 | 0.003 | 0.014 | 0.006 |  |  |  |  |  |  |  |  |  |  |  |  |  |  |  |  |  |  |  |  |  |
| PIX | **24** | 0.021 | 0.014 | 0.02 | 0.003 | 0.014 | 0.011 | 0.011 | 0.014 | 0.012 | 0.012 | 0.003 | 0.008 | 0.014 | 0.003 | 0.015 | 0.002 | 0.008 | 0.017 | 0.008 | 0.003 | 0.014 | 0.006 | 0.003 |  |  |  |  |  |  |  |  |  |  |  |  |  |  |  |  |  |  |  |  |
| PIX | **25** | 0.017 | 0.009 | 0.015 | 0.014 | 0.009 | 0.012 | 0.006 | 0.009 | 0.008 | 0.008 | 0.014 | 0.015 | 0.009 | 0.014 | 0.011 | 0.012 | 0.015 | 0.012 | 0.012 | 0.014 | 0.009 | 0.017 | 0.014 | 0.014 |  |  |  |  |  |  |  |  |  |  |  |  |  |  |  |  |  |  |  |
| FSC | **26** | 0.021 | 0.014 | 0.02 | 0.003 | 0.014 | 0.011 | 0.011 | 0.014 | 0.012 | 0.012 | 0.003 | 0.008 | 0.014 | 0.003 | 0.015 | 0.002 | 0.008 | 0.017 | 0.008 | 0.003 | 0.014 | 0.006 | 0.003 | 0.003 | 0.014 |  |  |  |  |  |  |  |  |  |  |  |  |  |  |  |  |  |  |
| FSC | **27** | 0.021 | 0.014 | 0.02 | 0.003 | 0.014 | 0.011 | 0.011 | 0.014 | 0.012 | 0.012 | 0.003 | 0.008 | 0.014 | 0.003 | 0.015 | 0.002 | 0.008 | 0.017 | 0.008 | 0.003 | 0.014 | 0.006 | 0.003 | 0.003 | 0.014 | 0.003 |  |  |  |  |  |  |  |  |  |  |  |  |  |  |  |  |  |
| FSC | **28** | 0.018 | 0.011 | 0.017 | 0.015 | 0.011 | 0.014 | 0.008 | 0.008 | 0.009 | 0.006 | 0.015 | 0.017 | 0.011 | 0.015 | 0.006 | 0.014 | 0.02 | 0.008 | 0.014 | 0.015 | 0.011 | 0.018 | 0.015 | 0.015 | 0.011 | 0.015 | 0.015 |  |  |  |  |  |  |  |  |  |  |  |  |  |  |  |  |
| GRW1 | **29** | 0.024 | 0.017 | 0.023 | 0.006 | 0.017 | 0.014 | 0.011 | 0.017 | 0.015 | 0.015 | 0.006 | 0.011 | 0.017 | 0.003 | 0.018 | 0.005 | 0.008 | 0.02 | 0.011 | 0.006 | 0.017 | 0.009 | 0.006 | 0.006 | 0.011 | 0.006 | 0.006 | 0.018 |  |  |  |  |  |  |  |  |  |  |  |  |  |  |  |
| GRW1 | **30** | 0.015 | 0.011 | 0.017 | 0.012 | 0.011 | 0.014 | 0.008 | 0.009 | 0.009 | 0.003 | 0.012 | 0.014 | 0.008 | 0.012 | 0.009 | 0.011 | 0.017 | 0.011 | 0.014 | 0.012 | 0.011 | 0.015 | 0.012 | 0.012 | 0.011 | 0.012 | 0.012 | 0.009 | 0.015 |  |  |  |  |  |  |  |  |  |  |  |  |  |  |
| GRW1 | **31** | 0.002 | 0.01 | 0.003 | 0.021 | 0.01 | 0.018 | 0.013 | 0.016 | 0.014 | 0.011 | 0.021 | 0.022 | 0.013 | 0.021 | 0.018 | 0.019 | 0.026 | 0.018 | 0.018 | 0.019 | 0.01 | 0.022 | 0.021 | 0.021 | 0.016 | 0.021 | 0.021 | 0.018 | 0.024 | 0.014 |  |  |  |  |  |  |  |  |  |  |  |  |  |
| GRW2 | **32** | 0.021 | 0.014 | 0.02 | 0.003 | 0.014 | 0.011 | 0.011 | 0.012 | 0.012 | 0.012 | 0.003 | 0.008 | 0.014 | 0.003 | 0.015 | 0.002 | 0.008 | 0.017 | 0.008 | 0.003 | 0.014 | 0.006 | 0.003 | 0.003 | 0.014 | 0.003 | 0.003 | 0.015 | 0.006 | 0.009 | 0.021 |  |  |  |  |  |  |  |  |  |  |  |  |
| GRW2 | **33** | 0.023 | 0.015 | 0.021 | 0.005 | 0.015 | 0.012 | 0.009 | 0.015 | 0.014 | 0.014 | 0.005 | 0.009 | 0.015 | 0.002 | 0.017 | 0.003 | 0.009 | 0.018 | 0.006 | 0.005 | 0.015 | 0.005 | 0.005 | 0.005 | 0.015 | 0.005 | 0.005 | 0.017 | 0.005 | 0.014 | 0.022 | 0.005 |  |  |  |  |  |  |  |  |  |  |  |
| GRW2 | **34** | 0.017 | 0.009 | 0.015 | 0.014 | 0.009 | 0.012 | 0.006 | 0.009 | 0.002 | 0.008 | 0.014 | 0.015 | 0.009 | 0.014 | 0.011 | 0.012 | 0.018 | 0.012 | 0.012 | 0.014 | 0.009 | 0.017 | 0.014 | 0.014 | 0.009 | 0.014 | 0.014 | 0.011 | 0.017 | 0.011 | 0.016 | 0.014 | 0.015 |  |  |  |  |  |  |  |  |  |  |
| GRW2 | **35** | 0.018 | 0.011 | 0.017 | 0.006 | 0.011 | 0.011 | 0.008 | 0.011 | 0.009 | 0.009 | 0.006 | 0.008 | 0.011 | 0.006 | 0.012 | 0.005 | 0.011 | 0.014 | 0.008 | 0.006 | 0.011 | 0.009 | 0.006 | 0.006 | 0.011 | 0.006 | 0.006 | 0.012 | 0.009 | 0.009 | 0.018 | 0.006 | 0.008 | 0.011 |  |  |  |  |  |  |  |  |  |
| FLW | **36** | 0.015 | 0.008 | 0.014 | 0.012 | 0.008 | 0.011 | 0.005 | 0.005 | 0.006 | 0.003 | 0.012 | 0.014 | 0.008 | 0.012 | 0.003 | 0.011 | 0.017 | 0.005 | 0.011 | 0.012 | 0.008 | 0.015 | 0.012 | 0.012 | 0.008 | 0.012 | 0.012 | 0.003 | 0.015 | 0.006 | 0.014 | 0.012 | 0.014 | 0.008 | 0.009 |  |  |  |  |  |  |  |  |
| FLW | **37** | 0.017 | 0.009 | 0.015 | 0.014 | 0.009 | 0.012 | 0.006 | 0.006 | 0.008 | 0.005 | 0.014 | 0.015 | 0.009 | 0.014 | 0.005 | 0.012 | 0.018 | 0.006 | 0.012 | 0.014 | 0.009 | 0.017 | 0.014 | 0.014 | 0.009 | 0.014 | 0.014 | 0.005 | 0.017 | 0.008 | 0.014 | 0.014 | 0.015 | 0.009 | 0.011 | 0.002 |  |  |  |  |  |  |  |
| FLW | **38** | 0.02 | 0.012 | 0.018 | 0.017 | 0.012 | 0.015 | 0.009 | 0.009 | 0.011 | 0.008 | 0.017 | 0.018 | 0.012 | 0.017 | 0.005 | 0.015 | 0.021 | 0.006 | 0.015 | 0.017 | 0.009 | 0.02 | 0.017 | 0.017 | 0.012 | 0.017 | 0.017 | 0.008 | 0.02 | 0.011 | 0.019 | 0.017 | 0.018 | 0.012 | 0.014 | 0.005 | 0.006 |  |  |  |  |  |  |
| FLW | **39** | 0.02 | 0.012 | 0.018 | 0.017 | 0.012 | 0.015 | 0.009 | 0.009 | 0.011 | 0.008 | 0.017 | 0.018 | 0.012 | 0.017 | 0.008 | 0.015 | 0.021 | 0.009 | 0.015 | 0.017 | 0.012 | 0.02 | 0.017 | 0.017 | 0.012 | 0.017 | 0.017 | 0.008 | 0.02 | 0.011 | 0.019 | 0.017 | 0.018 | 0.012 | 0.014 | 0.005 | 0.006 | 0.003 |  |  |  |  |  |
| FLW | **40** | 0.017 | 0.009 | 0.015 | 0.014 | 0.009 | 0.012 | 0.006 | 0.006 | 0.008 | 0.005 | 0.014 | 0.015 | 0.009 | 0.014 | 0.005 | 0.012 | 0.018 | 0.006 | 0.012 | 0.014 | 0.009 | 0.017 | 0.014 | 0.014 | 0.009 | 0.014 | 0.014 | 0.005 | 0.017 | 0.008 | 0.016 | 0.014 | 0.015 | 0.009 | 0.011 | 0.002 | 0.003 | 0.006 | 0.006 |  |  |  |  |
| FLW | **41** | 0.021 | 0.014 | 0.02 | 0.017 | 0.014 | 0.012 | 0.012 | 0.012 | 0.014 | 0.011 | 0.017 | 0.018 | 0.015 | 0.017 | 0.009 | 0.015 | 0.021 | 0.011 | 0.018 | 0.017 | 0.014 | 0.02 | 0.017 | 0.017 | 0.015 | 0.017 | 0.017 | 0.011 | 0.02 | 0.011 | 0.021 | 0.017 | 0.018 | 0.015 | 0.014 | 0.008 | 0.009 | 0.006 | 0.006 | 0.009 |  |  |  |
| VIG | **42** | 0.043 | 0.041 | 0.044 | 0.046 | 0.041 | 0.043 | 0.037 | 0.041 | 0.036 | 0.036 | 0.046 | 0.047 | 0.038 | 0.044 | 0.04 | 0.044 | 0.047 | 0.041 | 0.044 | 0.046 | 0.041 | 0.046 | 0.046 | 0.046 | 0.038 | 0.046 | 0.046 | 0.043 | 0.044 | 0.04 | 0.043 | 0.046 | 0.046 | 0.038 | 0.043 | 0.04 | 0.041 | 0.044 | 0.041 | 0.041 | 0.046 |  |  |
| VIG | **43** | 0.044 | 0.043 | 0.046 | 0.047 | 0.043 | 0.044 | 0.039 | 0.043 | 0.038 | 0.038 | 0.047 | 0.049 | 0.04 | 0.046 | 0.041 | 0.046 | 0.049 | 0.043 | 0.046 | 0.047 | 0.043 | 0.047 | 0.047 | 0.047 | 0.04 | 0.047 | 0.047 | 0.044 | 0.046 | 0.041 | 0.045 | 0.047 | 0.047 | 0.04 | 0.044 | 0.041 | 0.043 | 0.046 | 0.043 | 0.043 | 0.047 | 0.002 |  |
| VIG | **44** | 0.046 | 0.044 | 0.047 | 0.049 | 0.044 | 0.046 | 0.04 | 0.044 | 0.04 | 0.04 | 0.049 | 0.05 | 0.041 | 0.047 | 0.043 | 0.047 | 0.05 | 0.044 | 0.047 | 0.049 | 0.044 | 0.049 | 0.049 | 0.049 | 0.041 | 0.049 | 0.049 | 0.046 | 0.047 | 0.043 | 0.046 | 0.049 | 0.049 | 0.041 | 0.046 | 0.043 | 0.044 | 0.047 | 0.044 | 0.044 | 0.049 | 0.003 | 0.002 |

**Table S3 – Pairwise F_ST_ values between populations of Cingula trifasciata, based on the COI dataset.**

|  | **Santa Maria** | **Caloura** | **Mosteiros** | **Graciosa** | **Pico** | **São Jorge** | **Flores** | **Vigo** |
| --- | --- | --- | --- | --- | --- | --- | --- | --- |
| **Santa Maria** |  |  |  |  |  |  |  |  |
| **Caloura** | 0.222** |  |  |  |  |  |  |  |
| **Mosteiros** | 0.262** | 0.048 |  |  |  |  |  |  |
| **Graciosa** | 0.361** | 0.153** | 0.061 |  |  |  |  |  |
| **Pico** | 0.527** | 0.293 | 0.129 | 0.083 |  |  |  |  |
| **São Jorge** | 0.419** | 0.186 | 0.041 | 0.056 | -0.014 |  |  |  |
| **Flores** | 0.433** | 0.308** | 0.346** | 0.712** | 0.611** | 0.529** |  |  |
| **Vigo** | 0.855** | 0.852** | 0.848** | 0.869** | 0.944** | 0.929** | 0.949** |  |
| Significance tests derived from 1023 permutations; ** p<0.01. | | | | | | | | |

**Table S4 - Pairwise F_ST_ values between populations of *Cingula trifasciata*, based on the complete WAI dataset.**

|  | **Santa Maria** | **Caloura** | **Mosteiros** | **Graciosa** | **Pico** | **São Jorge** | **Vigo** |
| --- | --- | --- | --- | --- | --- | --- | --- |
| **Santa Maria** |  |  |  |  |  |  |  |
| **Caloura** | 0.177** |  |  |  |  |  |  |
| **Mosteiros** | 0.198** | 0.173** |  |  |  |  |  |
| **Graciosa** | 0.143** | 0.142** | 0.119** |  |  |  |  |
| **Pico** | 0.193** | 0.222** | 0.110** | 0.122** |  |  |  |
| **São Jorge** | 0.166** | 0.212** | 0.169** | 0.138** | 0.178** |  |  |
| **Vigo** | 0.359** | 0.382** | 0.430 ** | 0.393** | 0.442** | 0.365** |  |
| Significance tests derived from 1000 permutations; ** p<0.01. | | | | | | | |

**Table S5 - Estimates of evolutionary divergence (raw p-distances) between COI sequences of several Rissoidae species.** The analysis, performed on MEGA v7 [1], considered 18 sequences and 658 bp after excluding positions containing gaps and/or missing data for each sequence pair analysed. Sequences retrieved from GenBank [2], accession Number (AN) and species identification are provided in the table. Highlighted in bold is the divergence level between the recognized species *Alvania formicarum* and *A. mediolittoralis*, and between *Cingula trifasciata* from the Azores and Vigo.

| **Species** | **AN** | **1** | **2** | **3** | **4** | **5** | **6** | **7** | **8** | **9** | **10** | **11** | **12** | **13** | **14** | **15** | **16** | **17** |
| --- | --- | --- | --- | --- | --- | --- | --- | --- | --- | --- | --- | --- | --- | --- | --- | --- | --- | --- |
| Alvania angioyi | MG652373 |  |  |  |  |  |  |  |  |  |  |  |  |  |  |  |  |  |
| Alvania beani | FN650144 | 0.139 |  |  |  |  |  |  |  |  |  |  |  |  |  |  |  |  |
| Alvania formicarum | MG652380 | 0.144 | 0.125 |  |  |  |  |  |  |  |  |  |  |  |  |  |  |  |
| Alvania mediolittoralis | MG652385 | 0.144 | 0.123 | **0.029** |  |  |  |  |  |  |  |  |  |  |  |  |  |  |
| Alvania punctura | FN650148 | 0.141 | 0.153 | 0.178 | 0.172 |  |  |  |  |  |  |  |  |  |  |  |  |  |
| Alvania sleursi | MG652386 | 0.15 | 0.116 | 0.106 | 0.096 | 0.176 |  |  |  |  |  |  |  |  |  |  |  |  |
| Alvania subsoluta | FN650150 | 0.151 | 0.146 | 0.15 | 0.148 | 0.168 | 0.15 |  |  |  |  |  |  |  |  |  |  |  |
| Cingula trifasciata Azores | MG652397 | 0.128 | 0.152 | 0.147 | 0.147 | 0.15 | 0.152 | 0.137 |  |  |  |  |  |  |  |  |  |  |
| Cingula trifasciata Vigo | MW518858 | 0.125 | 0.149 | 0.140 | 0.146 | 0.160 | 0.158 | 0.145 | **0.044** |  |  |  |  |  |  |  |  |  |
| Crisilla postrema | MG652392 | 0.141 | 0.207 | 0.202 | 0.198 | 0.158 | 0.207 | 0.188 | 0.152 | 0.149 |  |  |  |  |  |  |  |  |
| Onoba semicostata | FN650151 | 0.176 | 0.177 | 0.17 | 0.167 | 0.201 | 0.176 | 0.162 | 0.173 | 0.176 | 0.199 |  |  |  |  |  |  |  |
| Pseudosetia sp. | FN650172 | 0.178 | 0.155 | 0.169 | 0.166 | 0.196 | 0.17 | 0.132 | 0.167 | 0.172 | 0.191 | 0.176 |  |  |  |  |  |  |
| Pseudosetia semipellucida | FN650154 | 0.202 | 0.182 | 0.187 | 0.193 | 0.187 | 0.191 | 0.174 | 0.191 | 0.148 | 0.199 | 0.187 | 0.179 |  |  |  |  |  |
| Pusillina inconspicua | FN650157 | 0.176 | 0.188 | 0.188 | 0.181 | 0.195 | 0.185 | 0.164 | 0.181 | 0.179 | 0.205 | 0.185 | 0.188 | 0.216 |  |  |  |  |
| Pusillina sarsii | FN650160 | 0.176 | 0.191 | 0.195 | 0.187 | 0.198 | 0.188 | 0.167 | 0.181 | 0.179 | 0.208 | 0.185 | 0.187 | 0.219 | 0.009 |  |  |  |
| Rissoa auriscalpium | HQ623175 | 0.197 | 0.206 | 0.202 | 0.193 | 0.204 | 0.201 | 0.215 | 0.213 | 0.218 | 0.221 | 0.204 | 0.208 | 0.215 | 0.199 | 0.208 |  |  |
| Rissoa guernei | MG652409 | 0.19 | 0.21 | 0.202 | 0.207 | 0.219 | 0.196 | 0.217 | 0.205 | 0.195 | 0.213 | 0.21 | 0.213 | 0.229 | 0.196 | 0.199 | 0.199 |  |
| Setia subvaricosa | MG652429 | 0.144 | 0.161 | 0.16 | 0.157 | 0.184 | 0.182 | 0.157 | 0.164 | 0.167 | 0.179 | 0.173 | 0.179 | 0.201 | 0.170 | 0.173 | 0.210 | 0.188 |

**
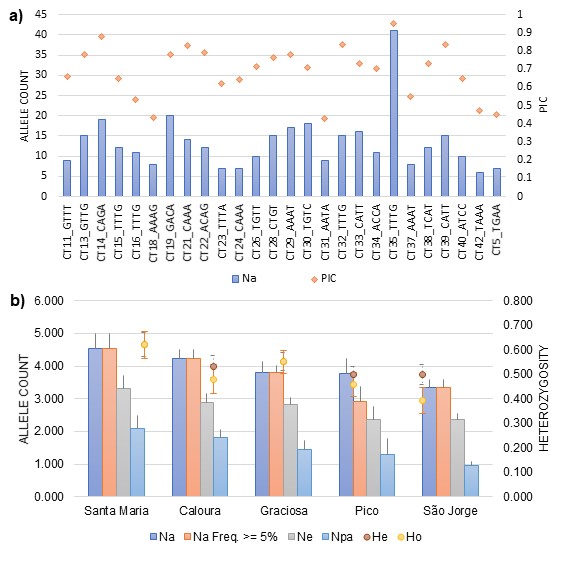
**

**Figure S 1 –** **Genetics diversity of the SSR dataset in study. a)** Variability measures estimated for each of the 26 SSR loci in study in complete whole amplicon information (WAI) dataset: number of alleles (Na) and polymorphic information content (PIC); **b)** Genetic diversity patterns across the five populations of *Cingula trifasciata* with more than five individuals: number of alleles (Na), number of different alleles with frequency over 5 %, number of effective alleles (Ne), number of private alleles (Npa), expected (*He*) and observed (*Ho*) heterozygosity.


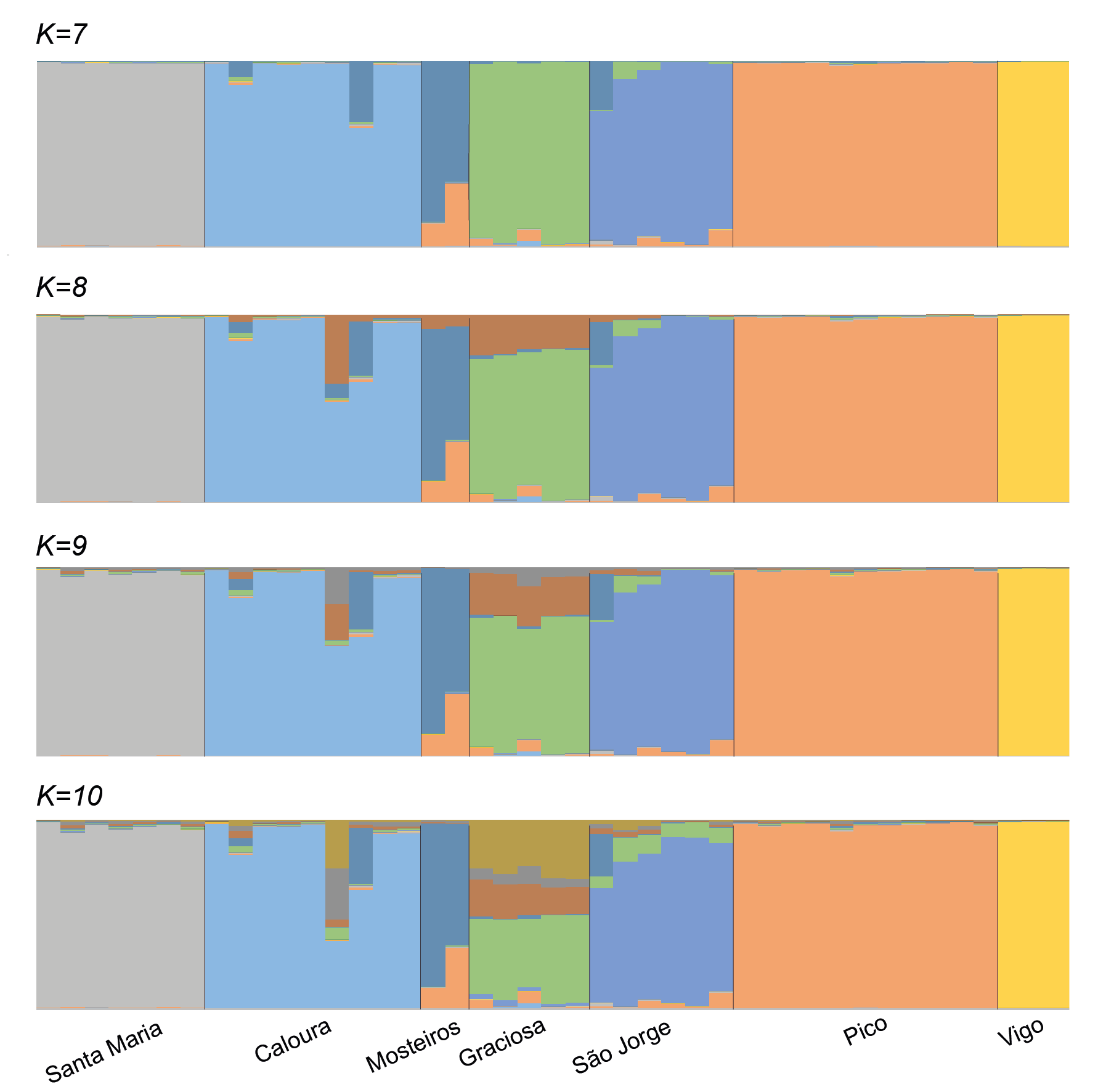


**Fig. S 2** – **Genetic structure analysis of the complete WAI dataset of *Cingula trifasciata*.** Inferred with STRUCTURE v2.3.4 [5,6], reporting the results from K=7 to K=10.

**References**

[1] Kumar S, Stecher G, Tamura K. MEGA7: Molecular Evolutionary Genetics Analysis version 7.0 for bigger datasets. Mol Biol Evol. 2016;33:1870–4. doi:10.1093/molbev/msw054.

[2] GenBank Database, National Center for Biotechnology Information. https://www.ncbi.nlm.nih.gov/genbank/. Accessed 5 May 2020.

[3] Peakall R, Smouse PE. GENALEX 6: genetic analysis in Excel. Population genetic software for teaching and research. Mol Ecol Notes. 2006;6:288–95. doi:10.1111/j.1471-8286.2005.01155.x.

[4] Peakall R, Smouse PE. GenAlEx 6.5: genetic analysis in Excel. Population genetic software for teaching and research-an update. Bioinformatics. 2012;28:2537–9. doi:10.1093/bioinformatics/bts460.

[5] Pritchard JK, Stephens M, Donnely P. Inference of population structure using multilocus genotype data. Genetics. 2000;155:945–59.

[6] Hubisz M, Falush D, Stephens M, Pritchard JK. Inferring weak population structure with the assistance of sample group information. Mol Ecol Res. 2009;9:1322–32. doi:10.1111/j.1755-0998.2009.02591.x.
